# Stereotactic arrhythmia radioablation for refractory ventricular tachycardia: A narrative review and pooled analysis of clinical outcomes and treatment delivery approaches

**DOI:** 10.1002/acm2.70622

**Published:** 2026-05-12

**Authors:** Keyur D. Shah, Chih‐Wei Chang, Sibo Tian, Pretesh Patel, Richard Qiu, Shadab Momin, Justin Roper, Jun Zhou, Zhen Tian, Xiaofeng Yang

**Affiliations:** ^1^ Department of Radiation Oncology and Winship Cancer Institute Emory University Atlanta Georgia USA; ^2^ Department of Radiation & Cellular Oncology University of Chicago Chicago Illinois USA

**Keywords:** cardiac radiosurgery, noninvasive VT ablation, stereotactic arrhythmia radioablation (STAR), stereotactic body radiation therapy (SBRT), ventricular tachycardia (VT)

## Abstract

**Purpose:**

Stereotactic arrhythmia radioablation (STAR) has emerged as a noninvasive salvage therapy for refractory ventricular tachycardia (VT), particularly in patients ineligible for catheter ablation (CA). This narrative review and pooled analysis evaluates STAR's efficacy, safety, and technical characteristics, integrating evidence from preclinical studies, case reports, case series, and clinical trials.

**Methods:**

A comprehensive literature search identified 88 studies published between 2015 and 2025, comprising 13 preclinical investigations, 50 case reports, 18 case series, and 7 clinical trials. Study‐level data were extracted for pooled analyses of 6‐ and 12‐month mortality, VT burden reduction, and Grade 3+ acute toxicity. Subgroup analyses were performed by delivery modality, age, left ventricular ejection fraction (LVEF), and cardiomyopathy type.

**Results:**

Pooled 6‐ and 12‐month mortality were 16% (95% CI: 11%–20%) and 33% (95% CI: 27%–38%), respectively. VT burden reduction at 6 months averaged 75% (95% CI: 73%–77%), with substantial heterogeneity (*I* ^2^ = 98.8%). Grade 3+ acute toxicity occurred in 7% (95% CI: 4%–10%), most commonly heart‐failure decompensation or pneumonitis. Subgroup analyses indicated more favorable outcomes in younger patients, non‐ischemic cardiomyopathy (NICM), and those with higher baseline LVEF.

**Conclusions:**

STAR achieves meaningful VT suppression with acceptable acute toxicity across multiple delivery modalities. However, wide inter‐study heterogeneity underscores the need for standardized endpoint definitions, dosimetric and motion‐management protocols, and prospective follow‐up registries. Translational preclinical data and emerging clinical evidence collectively support STAR's continued development as a biologically informed cardiac radiotherapy paradigm.

## 1 Introduction

Ventricular tachycardia (VT) is a life‐threatening arrhythmia characterized by rapid electrical activity originating in the ventricles, which can compromise cardiac output and lead to hemodynamic instability.^1^, ^2^ VT circuits are commonly associated with myocardial scar tissue, typically resulting from conditions such as myocardial infarction or cardiomyopathies.^3^, ^4^ The reentrant mechanism is a prevalent cause of VT, where electrical impulses circulate through areas of slow conduction, including the central isthmus, entrance, and exit sites, which sustain arrhythmic activity.^5^, ^6^ Between 2007 and 2020, VT was linked to over 7000 deaths in the United States among patients with underlying cardiovascular disease, underscoring the significant mortality burden associated with this arrhythmia.^7^

The management of VT includes pharmacological therapy, device‐based interventions, and catheter ablation (CA).^8^ Antiarrhythmic drugs such as amiodarone and beta‐blockers are first‐line treatments, but their efficacy is often limited, and they may carry significant side effects.^9^ Implantable cardioverter‐defibrillators (ICDs) are a cornerstone in preventing sudden cardiac death by terminating VT episodes through either anti‐tachycardia pacing or defibrillation shocks^10^; however, they do not prevent arrhythmia recurrence and can negatively impact the quality of life due to frequent shocks and complications.^11^

CA has emerged as a primary interventional strategy, aiming to eliminate arrhythmogenic substrates by using radiofrequency or cryothermal energy to create lesions that disrupt reentrant circuits.^12^ Despite its effectiveness, CA is invasive and may be challenging in patients with extensive myocardial scar burden or hemodynamic instability. Procedural success is highly dependent on precise identification and targeting of VT circuits, often guided by electroanatomical mapping (EAM), which integrates functional and anatomical data for improved precision. While CA is a primary interventional strategy for VT, it is not curative in all cases, particularly in patients with extensive scar burden or inaccessible arrhythmogenic tissue. Additionally, the procedure is associated with elevated risks of complications and mortality, especially in those with advanced heart failure or significant comorbidities.^13^, ^14^

Stereotactic arrhythmia radioablation (STAR) has emerged as a promising non‐invasive salvage option for treating VT in patients who are not candidates for or have failed CA. STAR offers a non‐invasive alternative for VT treatment, leveraging high‐dose radiation to modify arrhythmogenic substrates. Unlike catheter‐based interventions, STAR eliminates the risks of invasive procedures while providing precision targeting through advanced imaging modalities.^15^ The concept of using radiation therapy for VT management dates to the early 2000s in Japan. Miyashita et al.^16^ pioneered this approach by combining chemotherapy and RT to treat a case of right ventricular outflow tract (RVOT) VT in a 70‐year‐old female, delivering 40 Gy in a single fraction. This early effort was followed by Tanaka et al.,^17^ who applied a similar chemo‐RT regimen with 51 Gy in a single fraction to manage RVOT VT in a 65‐year‐old male. While these exploratory studies highlighted the potential of RT to address arrhythmic substrates, the lack of advanced imaging and delivery techniques limited the precision and safety of these early treatments.

The modern era of STAR began in 2015 when Loo et al.^18^ at Stanford demonstrated the first in‐human STAR procedure as it is known today. Using the CyberKnife system, they delivered 25 Gy in a single fraction to treat VT and achieved a remarkable 90.7% reduction in arrhythmic events. This groundbreaking work established the feasibility of non‐invasive VT ablation and paved the way for further exploration. In 2017, Cuculich et al.^19^ reported the first case series of STAR, treating five patients with 25 Gy in a single fraction and demonstrating an impressive 99.99% reduction in VT events. This study marked a pivotal moment in the field, showcasing STAR's clinical efficacy and safety.

Utilizing advanced imaging modalities and precision delivery platforms, STAR delivers highly conformal radiation doses to arrhythmogenic substrates. Its key advantages include non‐invasive delivery, outpatient feasibility, and the ability to target arrhythmogenic foci inaccessible by catheter‐based approaches. The growing body of evidence supports STAR as an effective option for reducing VT burden, with favorable acute and mid‐term outcomes. Most STAR treatments prescribe a dose of 25 Gy in a single fraction, guided by preclinical studies and clinical experience, with delivery platforms such as linear accelerators (LINACs), CyberKnife, and MRI‐guided systems offering unique capabilities in motion management and precision targeting.

Given the increasing adoption of STAR for VT management, there is a need for a comprehensive synthesis of the available evidence to assess its safety and efficacy. Several recent systematic reviews have summarized STAR outcomes, focusing on overall mortality, VT burden reduction, and early toxicity profiles.^20^, ^21^, ^22^, ^23^, ^24^ However, these studies have primarily pooled aggregate clinical endpoints and have not evaluated outcome heterogeneity across clinical subgroups—such as delivery modality, left ventricular ejection fraction (LVEF), age, or underlying cardiomyopathy type—or included translational findings from preclinical studies. This narrative review and pooled analysis address this gap by presenting the first exploratory subgroup synthesis of STAR outcomes stratified by patient and technical factors.

This narrative review and pooled analysis aims to:

- Summarize key findings from preclinical studies and case reports to provide translational and mechanistic context for STAR.
- Quantify mortality rates at 6‐ and 12‐months following STAR for VT in clinical cohorts.
- Assess the efficacy of STAR in reducing VT burden, including subgroup analyses based on delivery modality, patient characteristics, and cardiomyopathy type.
- Evaluate the incidence of acute grade 3+ toxicities associated with STAR in clinical studies.

This narrative review and pooled analysis includes data from preclinical studies, case reports, case series, and clinical trials, offering a comprehensive perspective on the evolving role of STAR in VT management. The findings aim to guide clinical practice and inform future research efforts to optimize patient selection, treatment planning, and posttreatment surveillance.

## 2 Materials and Methods

### 2.1 Search strategy and study selection

A structured literature search was conducted to identify relevant studies evaluating the use of radiation therapy for VT. The search was performed in PubMed, using the search term: “ventricular tachycardia AND radiation therapy.” The search covered studies published up to December 6, 2025, with filters applied to include specific study designs such as preclinical investigations, case reports, case series, and clinical trials. A total of 385 studies were initially identified through the database search. After the removal of duplicates, the remaining studies underwent screening based on title and abstract, followed by full‐text review.

Inclusion criteria were as follows:

- Studies reporting on preclinical investigations, case reports, case series, and clinical trials.
- Studies published in English.
- Studies reporting on distinct patient populations; in cases where multiple studies reported on the same cohort, the most recent publication was included.

Studies were excluded if they:

- Insufficient reporting on VT treatment using radiation therapy.
- Studies focused on unrelated topics, lacking clinical or preclinical relevance to radiation therapy for VT.
- Review articles, editorials, or commentaries that did not report original data.

**FIGURE 1:**
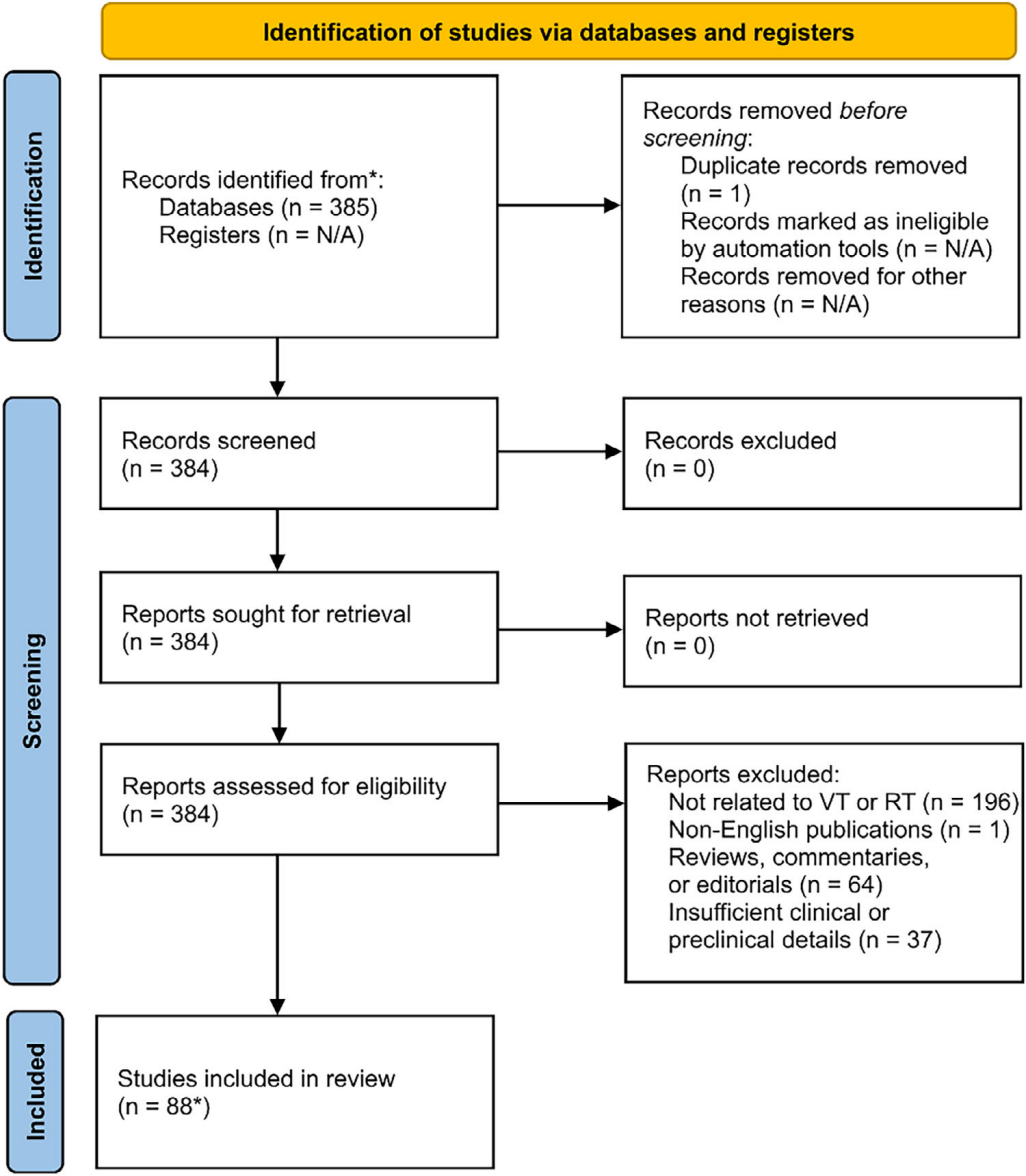
PRISMA flow diagram of study selection process. Illustrates the selection process for included studies, detailing the number of records identified, screened, excluded, and ultimately included in this review. Reasons for exclusion are categorized, including unrelated topics, insufficient data, duplicates, and non‐English publications. (*4 studies were added after independent citation analysis). Modified from Page et al.^25^ ^.^

Given the narrative and exploratory nature of this review, the search strategy was designed for breadth and relevance rather than exhaustive systematic retrieval. As such, the search strategy was not intended to meet formal systematic review standards but to capture representative and clinically relevant studies. Figure 1 illustrates the study selection process, detailing the number of records screened, included, and excluded at each stage.

### 2.2 Data extraction and analysis

Data were systematically extracted from the included studies, focusing on key parameters related to patient demographics, treatment characteristics, and clinical outcomes. The following variables were collected:

- Patient Characteristics: Sample size, median age, gender distribution, underlying cardiomyopathy (ischemic vs. non‐ischemic), median LVEF and prior CA history.
- Treatment Parameters: Radiation delivery modality (LINAC, CyberKnife, or MRI‐guided systems), prescribed dose, and planning target volume (PTV) margins.
- Clinical Outcomes: 6‐ and 12‐month mortality rates, VT burden reduction, adverse events (grade 3+ toxicity) within 90 days.

Adverse events were classified according to the Common Terminology Criteria for Adverse Events (CTCAE v5.0), where reported. For studies that did not specify the grading system, adverse events labeled as grade 3 or higher by the authors were included. When data were missing or ambiguously reported, the studies were excluded from the pooled analysis for that specific variable to maintain the robustness of results.

### 2.3 Statistical analysis

The pooled analysis employed a DerSimonian–Laird random‐effects model to account for inter‐study variability across the included clinical studies (specifically, case series and clinical trials). Single‐patient case reports were excluded from all quantitative pooled analyses to minimize bias and ensure methodological consistency. Key outcomes analyzed included mortality rates, efficacy measures, and safety profiles. Mortality rates were evaluated at 6 and 12 months. Subgroup analyses were conducted to examine differences based on cardiomyopathy type (ischemic vs. non‐ischemic), patient age (≤ median vs. > median), and LVEF (≤ median vs. > median). Age and LVEF subgrouping was performed using the median values reported at the study level. For each study, if the cohort‐level median (or mean, where median was unavailable) was above or below the pooled median across all studies, it was classified accordingly. For cardiomyopathy, patients were stratified based on studies that clearly separated ischemic (ICM) and non‐ischemic (NICM) etiologies; studies that reported mixed or ambiguous labeling were excluded from this subgroup analysis only, but were retained in the overall pooled analysis. Safety outcomes were reported as rates of grade 3 or higher adverse events occurring within 90 days of treatment. Treatment efficacy was assessed through pooled analysis of VT burden reduction, quantified as the percentage reduction in VT events at 6 months. Only studies that reported or enabled calculation of VT burden reduction over a 6‐month interval were included for this endpoint. VT burden was defined at the study level and included ICD‐treated VT episodes, sustained VT, or total arrhythmic events. Most estimates were derived from study‐level summaries (e.g., group means or medians); patient‐level data were not available. Blanking periods were variably applied and inconsistently reported across studies, contributing to outcome heterogeneity.

Heterogeneity across studies was assessed using the I‐squared (*I* ^2^) statistic and Cochran's Q test. *I* ^2^ values of 50% or higher were interpreted as indicative of moderate‐to‐high heterogeneity, and potential sources of heterogeneity were further explored through subgroup analyses. These subgroup analyses included comparisons between LINAC‐ and CyberKnife‐based treatments and evaluations of the impact of patient demographics, such as age and LVEF, on treatment outcomes.

All statistical analyses were performed using Python, utilizing the Statsmodels and SciPy libraries. Results were presented as pooled estimates with corresponding 95% confidence intervals (CIs).

## 3 Results

### 3.1 Study selection and characteristics

A total of 385 studies were reviewed, and 88 studies were included in this review, comprising 13 preclinical studies, 50 case reports, 18 case series, and 7 clinical trials, published between 2015 and 2025. The PRISMA flow diagram summarizing the study selection process is presented in Figure 1.

Studies were conducted across 19 countries, with the majority originating from Europe (*n* = 46), followed by North America (*n* = 23), Asia (*n* = 16) and Australia (*n* = 1). A total of 350 patients were analyzed across the included studies, with 89% male patients. Nearly 90% of the studies utilized 6 MV photons as the primary treatment modality, with 25 Gy prescribed as the standard dose to the arrhythmic substrate. The methods for defining target volumes varied across studies. However, the majority of studies (*n* = 85) incorporated cardiac computed tomography (CT) for anatomical localization and 12‐lead electrocardiography (ECG) for arrhythmic mapping. Advanced techniques such as EAM or electrocardiographic imaging (ECGI) were frequently employed (*n* = 78) to refine the target definition and delineate arrhythmogenic substrates.

Variability was observed in the reporting of outcomes, with some studies focusing on VT burden reduction and others prioritizing survival and toxicity as primary endpoints. Given the heterogeneity in study designs and follow‐up durations, pooled analyses with subgroup considerations (e.g., treatment modalities and motion management strategies) were conducted to provide a comprehensive assessment of outcomes.

### 3.2 Preclinical studies

A total of 13 preclinical studies explored the effects of STAR across diverse animal models, including pigs (*n* = 81), rabbits (*n* = 40), dogs (*n* = 25), and rats (*n* = 9), representing a combined total of 173 animals. These studies spanned five countries (USA, Japan, Germany, Russia, and South Korea) and provided critical insights into the efficacy and safety of STAR. The key characteristics and findings from these studies are summarized in Table 1. Studies assessed the impact of STAR on arrhythmia suppression, myocardial remodeling, conduction properties, and treatment safety. Photon‐based STAR was the most commonly studied modality in preclinical studies (38%, *n* = 5), reflecting its widespread clinical adoption for arrhythmia management across various settings. Particle therapy modalities, including protons and carbon ions, were equally studied, highlighting growing interest in their precision for treating arrhythmogenic substrates. These studies assessed the impact of radiation on arrhythmia management, myocardial remodeling, and treatment safety. Amino et al.^26^, ^27^ demonstrated dose‐dependent reductions in VT/VF inducibility, with higher doses leading to improved conduction parameters. Lehmann et al.^28^, ^29^ focused on achieving complete AV block with escalating doses, while Zei et al.^30^ reported successful electrical isolation of the RSPV, emphasizing the impact of STAR on conduction pathways and arrhythmia suppression. Structural studies, such as those by Hohmann et al.^31^, ^32^ and Kancharla et al.,^33^ highlighted enhanced scar homogenization and stabilization of cardiac function post‐MI. Molecular analysis from Kim et al.^34^ provided insights into early proteomic changes linked to radiation‐induced stress responses.

**TABLE 1:** Summary of preclinical studies investigating STAR and particle therapy for cardiac applications.

Authors (Year)	Country	Animal (n)	Disease model	Modality (Dose Gy)	Key findings
Lehmann et al. (2015)^28^	USA/Germany	Pigs (4)	Explanted	Carbons (70/90/160 Gy)	No AV block up to 130 Gy; complete AV block at 160 Gy, confirmed by PET‐CT; no visible myocardial damage.
Amino et al. (2017)^26^	Japan	Dogs (8)	AF	Carbons (15 Gy)	VT/VF inducibility reduced (25% vs. 100%) (irradiated (*n* = 4) vs non‐irradiated (*n* = 4)); improved conduction (QRS & RMS40); increased Cx43 (24%–45%).
Lehmann et al. (2017)^29^	USA	Pigs (10)	AV junction ablation	Photons (25/40/50/55 Gy)	Complete AV block achieved in 6/7 irradiated pigs (86%); lesion size increased with dose; no short‐term side effects; no damage to esophagus, phrenic nerves, or trachea; histology revealed beam effects outside target volume.
Zei et al. (2018)^30^	USA	Dogs + Pigs (19)	VT	Photons (15/20/25/35 Gy)	Successful electrical isolation of the RSPV achieved at 25 and 35 Gy (100%), partial isolation at 20 Gy (80%) and 15 Gy (50%); no complications or collateral tissue injury; transmural scar formation confirmed by histopathology.
Hohmann et al. (2019)^31^	USA	Pigs (20)	LV ablation (Healthy)	Protons (30/40 Gy)	Dose‐dependent decline in LVEF (*r* = ‐0.69, *P* = .008); LV dilation correlated with dose (*r* = 0.75, *P* = .003); functional decline observed ∼3 months posttreatment.
Hohmann et al. (2020)^32^	USA	Pigs (14)	Post‐MI	Protons (30/40 Gy)	Scar homogenization (treated: 30.1% myocytes vs. untreated: 59.9%); 4 VT‐related sudden deaths; stable cardiac function; MRI revealed dose‐related tissue effects over time.
Takami et al. (2021)^35^	Japan	Rabbits (32)	Whole LV irradiation	Carbons + Protons (25 Gy)	Significant LV conduction delays (PR: PT25 > control, *P* = .003); reduced *P* and QRS voltages; sustained effects at 6 months; VF induced in 1 carbon beam rabbit; no VF in proton group; mild‐moderate pericardial effusion in 19% (carbon) and 44% (proton) with no tamponade.
Vaskovskii et al. (2022)^37^	Russia	Pigs (2)	AV node and LV ablation	Photons (40/45 Gy)	40 Gy induced transient AV block; 45 Gy resulted in permanent AV block and ventricular standstill by day 21; histology confirmed transmurality and precision.
Kim et al. (2022)^34^	South Korea	Rats (9)	Proteomic (Healthy)	Photons (0/2/25 Gy)	25 Gy induced significant proteomic changes within 7 days; early effects on signal transduction, adhesion, and stress response; upregulation of oxidative stress proteins; potential mediators of early anti‐arrhythmic effects identified.
Amino et al. (2023)^27^	Japan	Rabbits (26)	HC, AT/AF & VT/VF	Carbons (15 Gy)	Radiation reduced AT/AF (1.2% vs. 9.9%) and VT/VF (1.2% vs. 7.8%); improved conduction velocity; reversed Cx40/43 downregulation and sympathetic nerve sprouting.
Imamura et al. (2023)^36^	USA	Pigs (19)	Normal + infarcted myocardium	Protons (40 Gy)	Reduced bipolar voltage amplitude (normal: 10.1→5.7 mV, infarcted: 2.0→0.8 mV); conduction velocity decreased (normal: 85→55 cm/s, infarcted: 43.7→26.3 cm/s); Cx43 reduction observed from 1‐week post‐irradiation; myocytolysis, capillary hyperplasia, and dilation at 8 weeks.
Kancharla et al. (2024)^33^	USA	Pigs (10)	Post‐MI VA	Photons (25 Gy)	SBRT reduced VA inducibility (100% vs. 25%, *P* = 0.07); scar density increased (33% vs. 14%, *P* = 0.07); no fibrosis in remote myocardium; SBRT improved scar homogenization.
Amino et al. (2025)^38^	Japan	Rabbits (8)	Hypercholesterolemia	Carbons (15 Gy)	PET imaging showed partial recovery of mitochondrial function and restored sympathetic nerve activity in irradiated hypercholesterolemic hearts, confirmed by tyrosine‐hydroxylase staining.

Abbreviation: AF, Atrial Fibrillation; AT, Atrial Tachycardia; AV, Atrioventricular; Cx40, Connexin‐40; Cx43, Connexin‐43; HC, Hypercholesterolemia; LVEF, Left Ventricular Ejection Fraction; MI, Myocardial Infarction; MRI, Magnetic Resonance Imaging; PET‐CT, Positron Emission Tomography‐Computed Tomography; PR, PR Interval; PT25, Proton Therapy 25 Gy; QRS, QRS Complex (ventricular depolarization); RMS40, Root Mean Square Voltage of the Last 40 ms; RSPV, Right Superior Pulmonary Vein; SBRT, Stereotactic Body Radiation Therapy; VT/VF, Ventricular Tachycardia/Fibrillation.

While STAR demonstrated promising efficacy, safety concerns were noted in specific studies. Takami et al.^35^ reported pericardial effusion in irradiated rabbits, while Imamura et al.^36^ observed conduction slowing and structural remodeling in long‐term follow‐up. Studies such as Vaskovskii et al.^37^ explored photon therapy's impact on AV node ablation, demonstrating dose‐dependent conduction block effects. These findings emphasize the need for precise dose optimization.

### 3.3 Case reports

The 50 included case reports, published between 2015 and 2025, provided detailed insights into individual patient experiences with STAR for recurrent VT. These studies predominantly utilized photon‐based STAR (*n* = 48), with 25 Gy in a single fraction being the standard dose prescription. Technologies used included LINAC systems, CyberKnife, and MRI‐guided systems, with a variety of motion management strategies such as 4DCT, internal target volume (ITV) expansion, and fiducial marker‐based tracking (e.g., using implanted radiopaque markers for real‐time respiratory motion tracking).

Most cases targeted monomorphic VT (MMVT) (*n* = 38), while polymorphic VT (PMVT) was less commonly reported (*n* = 12). PTV volumes varied significantly across cases, reflecting differences in arrhythmogenic substrate sizes and target delineation strategies. Notably, motion management techniques were adapted based on the technology used, with CyberKnife treatments employing fiducial markers and LINAC systems relying on 4DCT and ITV expansion.

Outcomes from these reports highlighted the efficacy of STAR in reducing VT burden (96.73% ± 6.41%), often achieving substantial suppression of arrhythmic episodes. While acute toxicities were rare, a few patients experienced pneumonitis or exacerbation of chronic obstructive pulmonary disease (COPD). However, long‐term follow‐up data were inconsistently reported, limiting the ability to draw definitive conclusions about the incidence and severity of late effects. A detailed summary of individual case reports, including treatment characteristics, is provided in Table 2.

**TABLE 2:** Summary of case reports investigating STAR for VT.

Author (Year)	Country	Age (Gender)	VT Type	Modality (Dose, fx)	Technology	PTV Volume (cc)	Motion management
Loo et al. (2015)^18^	USA	71 (M)	MMVT	Photons (25 Gy, 1fx)	CyberKnife	NR	Fiducial Marker
Jameau et al. (2018)^39^	Switzerland	75 (M)	PMVT	Photons (25 Gy, 1fx)	CyberKnife	21	Fiducial Marker
Haskova et al. (2018)^40^, ^41^	Czech Republic	34	PMVT	Photons (25 Gy, 1fx)	CyberKnife	62.2	NR
Bhaskaran et al. (2019)^42^	Canada	34 (F)	MMVT	Photons (25 Gy, 1fx)	LINAC	52	4DCT, ITV
Zeng et al. (2019)^43^	China	29 (M)	PMVT	Photons (25 Gy, 1fx)	CyberKnife	71.22	NR
Marti’‐Almor et al. (2020)^44^	Spain	64 (M)	MMVT	Photons (25 Gy, 1fx)	LINAC	NR	4DCT
Narducci et al. (2020)^45^	Italy	60 (M)	MMVT	Photons (25 Gy, 1fx)	LINAC	303	4DCT, ITV
Mayinger et al. (2020)^46^	Switzerland	71 (M)	MMVT	Photons (25 Gy, 1fx)	MRIdian	115.1	NR
Krug et al. (2020)^47^	Germany	78 (M)	MMVT	Photons (25 Gy, 1fx)	LINAC	42.2	NR
Park and Choi (2020)^48^	South Korea	76 (M)	MMVT	Photons (25 Gy, 1fx)	LINAC	NR	NR
Dusi et al. (2021)^49^	Italy	73 (M)	MMVT	Photons (25 Gy, 1fx)	NR	27.7	NR
Peichl et al. (2021)^41^, ^50^	Czech Republic	66 (M)	MMVT	Photons (25 Gy, 1fx)	CyberKnife	18.3	NR
Amino et al. (2021)^51^	Japan	75 (F)	PMVT	Photons (25 Gy, 1fx)	LINAC	49.7	NR
Quick et al. (2021)^52^	Germany	85 (M)	MMVT	Photons (25 Gy, 1fx)	NR	8.51, 15.01	NR
Lee et al. (2021)^53^	Korea	11 (M)	MMVT	Photons (25 Gy, 1fx)	LINAC	NR	4DCT, ITV
Kautzner et al. (2021)^54^	Czech Republic	52 (M)	MMVT	Photons (25 Gy, 1fx)	LINAC	52	NR
		57 (M)	MMVT	Photons (25 Gy, 1fx)	LINAC	62.1	NR
		67 (M)	PMVT	Photons (25 Gy, 1fx)	LINAC	70	NR
Thosani et al. (2021)^55^	USA	73 (M)	MMVT	Photons (25 Gy, 1fx)	LINAC	62.6	Margin
Aras et al. (2021)^56^	Turkey	58 (M)	MMVT	Photons (25 Gy, 1fx)	LINAC	NR	4DCT, ITV
Li et al. (2022)^57^	China	54 (M)	MMVT	Photons (25 Gy, 1fx)	LINAC	74.7	4DCT, ITV
Hayase et al. (2022)^58^	USA	78 (M)	MMVT	Photons (25 Gy, 1fx)	LINAC	NR	NR
Levis et al. (2022)^59^	Italy	73 (M)	MMVT	Photons (25 Gy, 1fx)	LINAC	89	4DCT
Haskova et al. (2022)^41^	Czech Republic	77 (M)	NR	Photons (25 Gy, 1fx)	CyberKnife	14.3	NR
Huang et al. (2022)^60^	Taiwan	63 (M)	MMVT	Photons (12 Gy, 1fx)	LINAC	65.75	ITV
van der Ree et al. (2022)^61^	Netherlands	60 (M)	PMVT	Photons (25 Gy, 1fx)	LINAC	300	4DCT, ITV
Wutzler et al. (2022)^62^	Germany	56 (M)	PMVT	Photons (25 Gy, 1fx)	LINAC	NR	4DCT, ITV
Bernstein et al. (2022)^63^	USA	75 (M)	MMVT	Photons (25 Gy, 1fx)	LINAC	87.9	4DCT
Kurzelowski et al. (2022)^64^	Poland	69 (M)	MMVT	Photons (25 Gy, 1fx)	LINAC	56.37	DIBH
		72	MMVT	Photons (25 Gy, 1fx)	LINAC	56.72	DIBH
Cybulska et al. (2022)^65^	Poland	67 (M)	PMVT	Photons (25 Gy, 1fx)	LINAC	NR	DIBH
Ninni et al. (2022)^66^	France	42 (M)	MMVT	Photons (25 Gy, 1fx)	CyberKnife	NR	NR
Nasu et al. (2022)^67^	Japan	58 (M)	PMVT	Photons (25 Gy, 1fx)	LINAC	29.1	4DCT
Pavone et al. (2022)^68^	Italy	73 (M)	PMVT	Photons (25 Gy, 1fx)	LINAC	NR	4DCT, ITV
Cozzi et al. (2022)^69^	Italy	81 (M)	MMVT	Photons (25 Gy, 1fx)	LINAC	122.5	4DCT, ITV
Mehrhof et al. (2023)^70^	Germany	54 (M)	MMVT	Photons (25 Gy, 1fx)	CyberKnife	75.2	Fiducial Marker
		61 (M)	PMVT	Photons (25 Gy, 1fx)	LINAC	134.6	ITV
Jiwani et al. (2023)^71^	USA	83 (M)	MMVT	Photons (25 Gy, 1fx)	LINAC	146.7	4DCT
van der Ree et al. (2023)^72^	Netherlands	47 (F)	PMVT	Photons (2 Gy, 2fx; 20 Gy, 1fx)	CyberKnife	16	Fiducial Tracking
Kaestner et al. (2023)^73^	Germany	63 (F)	MMVT	Photons (25 Gy, 1fx)	LINAC	NR	NR
Wijesuriya et al. (2023)^74^	UK	69 (F)	MMVT	Photons (25 Gy, 1fx)	LINAC	NR	NR
Vaskovskii et al. (2023)^75^	Russia	57 (M)	MMVT	Photons (25 Gy, 1fx)	LINAC	46	4DCT, ITV
Vozzolo et al. (2023)	USA	44 (M)	MMVT	Photons (25 Gy, 1fx)	LINAC	NR	4DCT
Keyt et al. (2023)^76^	USA	75 (M)	MMVT	Photons (25 Gy, 1fx)	LINAC	85	4DCT, ITV
Amino et al. (2024)^77^	Japan	60 (M)	MMVT	Carbons (25 Gy, 1fx)	XiO (Elekta)	29.7	Motion Margin
Kautzner et al. (2024)^78^	Czech Republic	54 (F)	MMVT	Photons (25 Gy, 1fx)	CyberKnife	NR	NR
Kaya et al. (2024)^79^	Netherlands	72 (M)	MMVT	Photons (25 Gy, 1fx)	LINAC	11^*^	4DCT
Trinh et al. (2025)^80^	USA	62 (M)	MMVT	Photons (25 Gy, 1fx)	LINAC	NR	NR
Cravéreau et al. (2025)^81^	France	54 (M)	MMVT	Photons (25 Gy, 1fx)	CyberKnife	383.18	4DCT, ITV

Abbreviations: 4DCT, Four‐Dimensional Computed Tomography; DIBH, Deep Inspiration Breath Hold; ITV, Internal Target Volume; LINAC, Linear Accelerator; MMVT, Monomorphic Ventricular Tachycardia; NR, Not Reported; PMVT, Polymorphic Ventricular Tachycardia.

*indicates Clinical Target Volume (CTV).

### 3.4 Clinical series: Case series and clinical trials

A total of 24 clinical trials and case series were included, with sample sizes ranging from 3 to 36 patients. These studies provided valuable insights into the efficacy of STAR for recurrent VT in larger cohorts. Across these studies, PTV values varied significantly, with a median of 81.55 cc (range: 14–372 cc), reflecting variability in target delineation practices and arrhythmogenic substrate sizes. Margins used for target volume expansion were inconsistent, ranging from 1 to 8 mm isotropic expansion, further emphasizing the variability in contouring practices across institutions.

Most studies employed photon‐based STAR, with doses predominantly prescribed at 25 Gy in a single fraction (*n* = 22, 91.7%). Of these, LINAC‐based systems were used in 79.2% of cases (*n* = 19), while CyberKnife treatments were reported in 16.7% (*n* = 4). One study used MRI‐guided STAR system, showcasing emerging technology for arrhythmia ablation.

Patient characteristics revealed significant baseline cardiac dysfunction, with a median LVEF of 27.0% (range: 10%–72%). Ischemic cardiomyopathy (ICM) and non‐ischemic cardiomyopathy (NICM) were nearly equally distributed, representing 51.81% and 48.19% of patients, respectively. Motion management techniques included 4DCT, ITV expansion, and fiducial marker‐based tracking, typically using implanted radiopaque markers (e.g., temporary pacing leads or ICD lead tips) to enable real‐time respiratory motion tracking via systems such as Synchrony. 4DCT was the most frequently employed strategy (62.5%), particularly in LINAC‐based treatments, while fiducial tracking was utilized for CyberKnife treatments. However, details on motion management were inconsistently reported in some studies, limiting the ability to evaluate specific trends.

On average, a 75.0% reduction in VT burden was observed at six months, highlighting the substantial arrhythmic suppression achieved with STAR. Details of the included clinical trials and case series, including patient demographics, cardiomyopathy classification, and treatment characteristics, are summarized in Table 3.

**TABLE 3:** Summary of clinical trials and case series investigating STAR for VT. If the entry is a clinical trial, its trial name is reported in the Author column.

Author (Year)	Country or region	Sample size	Age (Median, Range)	Gender (M/F)	CM	LVEF (%) (Median, Range)	PTV volume (cc)	Modality (Dose, fx)	Technology
Cuculich et al. (2017)^19^	USA	5	62 (60–83)	4 M/1F	2 ICM; 3 NICM	22 (15–26)	51.3 (17.3‐81)	Photons (25 Gy, 1fx)	LINAC
Robinson et al. (2019) (ENCORE‐VT)^82^	USA	19	66 (49–81)	17 M/2F	11 ICM; 8 NICM	25 (15–58)	98.9 (60.9‐298.8)	Photons (25 Gy, 1fx)	LINAC
Chin et al. (2020)^83^	USA	8	74 (65–86)	8 M	4 ICM; 4 NICM	20 (15–32.5)	84.9 (21.1–190.7)	Photons (15‐25 Gy, 1fx)	LINAC
Gianni et al. (2020)^84^	USA	5	67 (45–76)	5 M	4 ICM; 1 NICM	25 (20–55)	173 (80–184)	Photons (25 Gy, 1fx)	CyberKnife
Lee et al. (2021)^85^	UK	7	70 (60–79)	4 M/3F	5 ICM; 2 NICM	25 (15–45)	89.5 (57.5–139)	Photons (25 Gy, 1fx)	LINAC
Yugo et al. (2021)^86^	Taiwan	3	68 (65–83)	2 M/1F	3 NICM	44 (20–59)	70 (20–130)	Photons (25 Gy, 1fx)	LINAC
Ho et al. (2021)^87^	USA	6	72.5 (64–77)	6 M	2 ICM; 4 NICM	26 (10–46)	120.5 (66–193)	Photons (25 Gy, 1fx)	LINAC
Carbucicchio et al. (2021) (STAR‐MI‐VT)^88^	Italy	7	72 (59–78)	7 M	3 ICM; 4 NICM	21.1 (20.3–44.4)	198.3 (88.1–239)	Photons (25 Gy, 1fx)	LINAC
Qian et al. (2022)^89^	USA	6	72 (70–73)	6 M	6 ICM	20 (16–20)	319 (280–330)	Photons (25 Gy, 1fx)	LINAC
Wight et al. (2022)^90^, ^91^	USA	14	60.5 (50–70)	10 M/4F	5 ICM; 9 NICM	NR	NR	Photons (25 Gy, 1fx)	LINAC
Molon et al. (2022)^92^	Italy	6	79.5 (61–85)	5 M/1F	3 ICM; 3 NICM	26.5 (20–42)	NR	Photons (25 Gy, 1fx)	LINAC
Ninni et al. (2022)^93^	France	17	68 (30–83)	13 M/4F	10 ICM; 7 NICM	35 (20–53)	53.3 (19.96–185.88)	Photons (25 Gy, 1fx)	CyberKnife
Chang et al. (2023)^94^	Korea	6	72 (63–85)	4 M/2F	3 ICM; 3 NICM	31.5 (24–57)	52.2 (17.5–246.8)	Photons (25 Gy, 1fx)	LINAC
Aras et al. (2023)^95^	Turkey	8	61.5 (33–85)	8 M	2 ICM; 6 NICM	25 (10–30)	157.4 (70.5‐272.7)	Photons (25 Gy, 1fx)	LINAC
van der Ree et al. (2023) STARNL‐1^96^	Netherlands	6	73 (54–83)	6 M	6 ICM	38 (24–52)	187 (93–372)	Photons (25 Gy, 1fx)	LINAC
Krug et al. (2023) RAVENTA^97^	Germany	5	67 (49–74)	4 M/1F	2 ICM; 3 NICM	35 (20–45)	69.6 (43.4–80.7)	Photons (25 Gy, 1fx)	NR
Herrera Siklody et al. (2023)^98^	Switzerland	20	68 (47–80)	15 M/5F	6 ICM; 14 NICM	31 (20–72)	23 (14–115)	Photons (20‐25 Gy, 1fx)	CyberKnife/ MRIdian/ LINAC
Miszczyk et al. (2023) SMART‐VT^99^	Czech Republic	11	67 (45–72)	10 M/1F	9 ICM; 2 NICM	27 (20–40)	73 (18.6–111.3)	Photons (25 Gy, 1fx)	LINAC
Amino et al. (2023) (SRAT)^100^	Japan	3	71 (60–91)	1 M/2F	1 CIM; 2 NICM	27 (20–65)	55 (49.7–96.4)	Photons (25 Gy, 1fx)	LINAC
Haskova et al. (2024)^101^	Czech Republic	36	66 (56–76)	33 M/3F	20 ICM; 16 NICM	31 (22,40)	39.4 (12.6–90.5)	Photons (25 Gy, 1fx)	CyberKnife
Arkles et al. (2024)^102^	USA	15	65 (57.2–72.8)	13 M/2F	7 ICM; 8 NICM	30.2 (26.6–33.8)	45.6 (84.7–124.1)	Photons (25 Gy, 1fx)	LINAC
Borzov et al. (2024)^103^	Israel	3	64 (63–72)	3 M	1 ICM; 2 NICM	27.5 (15–30)	49.7 (47.8–91.8)	Photons (25 Gy, 1fx)	LINAC
Bianchi et al. (2024)^104^	Italy	11	68 (53–81)	11 M	5 ICM; 6 NICM	40 (30–57)	90.4 (30.6–119.5)	Photons (25 Gy, 1fx)	MRIdian
Das et al. (2025)^105^	Australia	12	74.9 (63.5–86.1)	10 M/2F	9 ICM; 3 NICM	20 (15.3–31.5)	135.1 (27.4–226.5)	Photons (25 Gy, 1fx)	LINAC

Abbreviations: CM, Cardiomyopathy; CyberKnife, Robotic Radiosurgery System; ICM, Ischemic Cardiomyopathy; LINAC, Linear Accelerator; LVEF, Left Ventricular Ejection Fraction; MRIdian, MRI‐Guided Radiation Therapy System; NICM, Non‐Ischemic Cardiomyopathy; NR, Not Reported; PTV, Planning Target Volume; fx, Fraction(s).

### 3.5 Pooled‐analysis

#### 3.5.1 6‐month and 12‐month mortality

**FIGURE 2:**
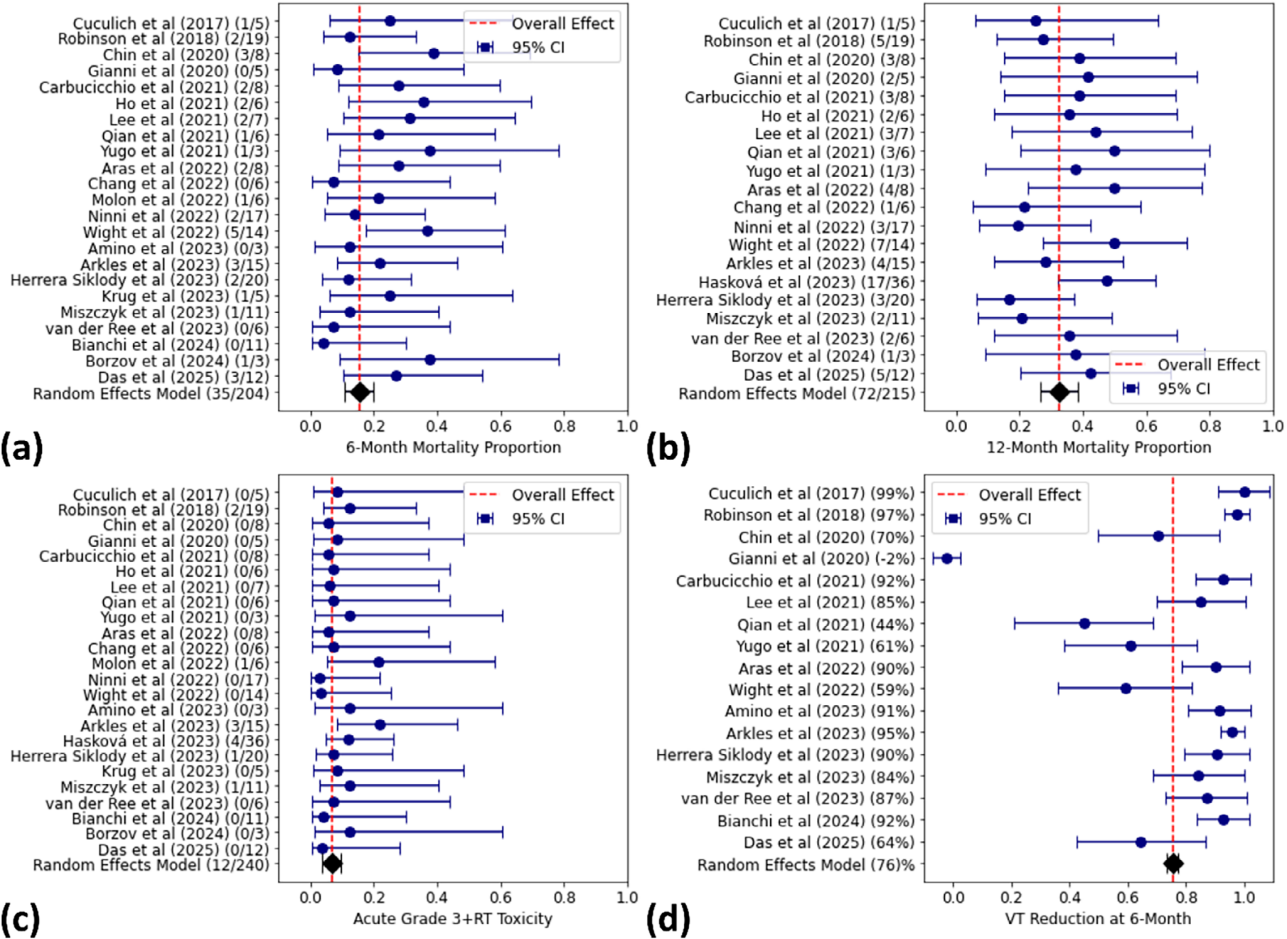
Forest Plots Summarizing Pooled‐Analysis Results for Mortality, VT Burden Reduction, and Acute Toxicity. Forest plots displaying the pooled effect estimates and 95% confidence intervals (CI) for (a) 6‐month mortality, (b) 12‐month mortality, (c) acute grade 3+ toxicity rates within 90 days, and (d) VT reduction at 6 months. The red dashed line represents the overall effect, while individual blue points and bars represent study‐specific estimates and their CIs. A random‐effects model was used for pooled‐analysis.

The pooled proportion of deaths at 6 months was 15.5% (95% CI: 11–20%), with minimal heterogeneity across studies (*I* ^2^ = 0.00%, Cochran's *Q* = 18.6, *p* = 0.67). For 12‐month mortality, the pooled estimate was 32.5% (95% CI: 26.7‐38.3%), also demonstrating low heterogeneity (*I* ^2^ = 0.00%, Cochran's *Q* = 16.47, *p* = 0.63). Figures 2(a) and 2(b) present the forest plots for 6‐month and 12‐month mortality, respectively.

#### 3.5.2 Grade 3 ± acute toxicities

The pooled rate of grade 3+ adverse events within 90 days of treatment was 7.2% (95% CI: 4.2%–10.3%), with no observed heterogeneity (*I* ^2^ = 0.00%, Cochran's *Q* = 7.6, *p* = 0.99). Toxicities included heart failure (*n* = 3), and esophagitis (*n* = 2). The forest plot for acute toxicity rates is shown in Figure 2(c).

#### 3.5.3 VT events reduction at 6 months

The pooled percentage reduction in VT events at 6 months was 75.4% (95% CI: 73.4%–77.4%), with substantial heterogeneity (*I* ^2^ = 98.80%, Cochran's *Q* = 1328.4, *p* < 0.05). Figure 2(d) illustrates the forest plot for VT event reduction. Notably, one study (Gianni *et al.* ^84^) demonstrated outlier behavior with markedly lower VT burden reduction compared to the majority of reports, which may reflect differences in baseline arrhythmic burden, endpoint definitions, or follow‐up protocols rather than intrinsic differences in treatment efficacy.

#### 3.5.4 Subgroup analysis

**FIGURE 3:**
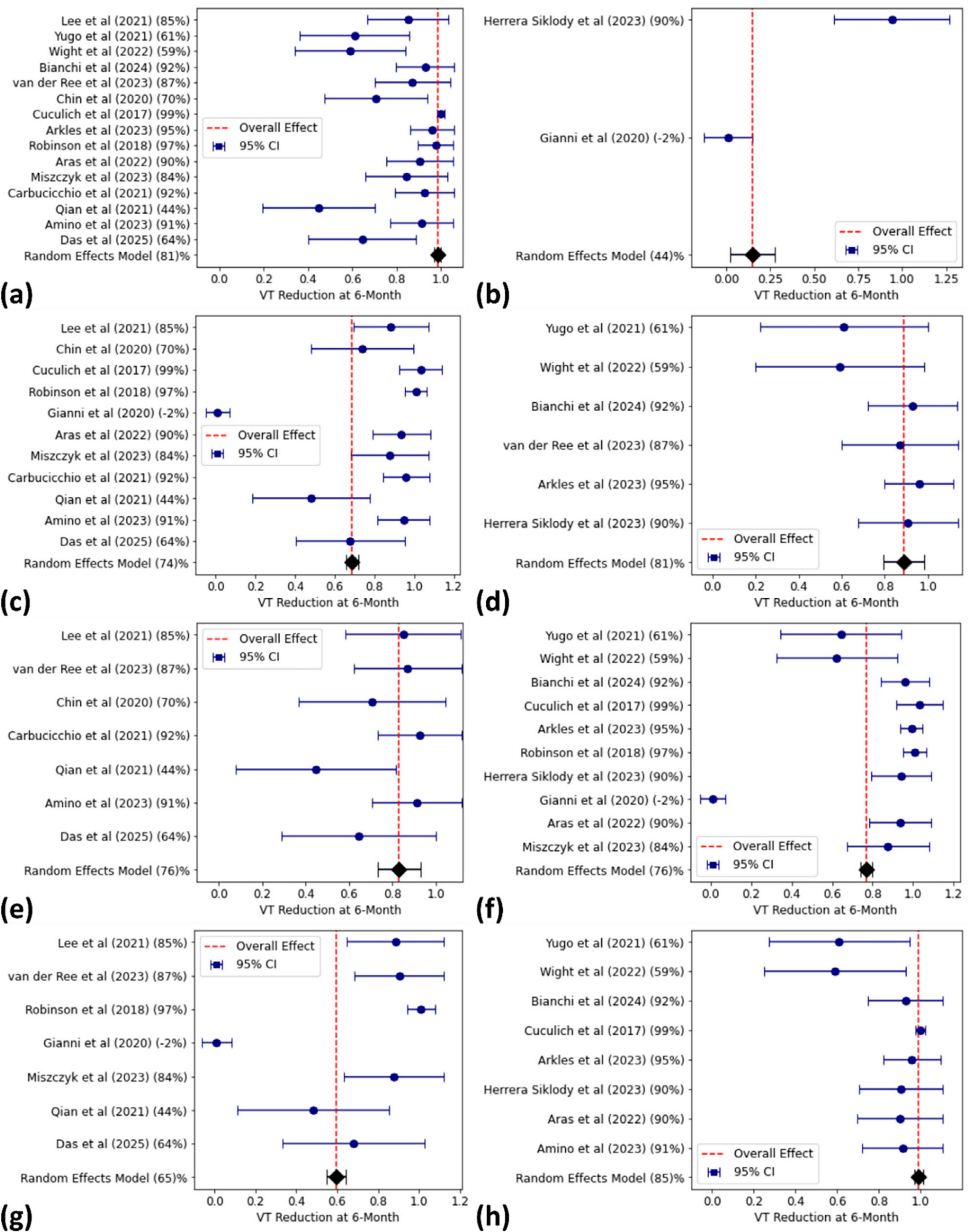
Forest plots depicting pooled VT burden reduction at 6 months following STAR, stratified by key subgroups. Each plot shows individual study estimates with 95% confidence intervals and an overall pooled estimate using a random‐effects model. Studies are labeled with their reported VT reduction (%) and ordered chronologically. Subgroup comparisons include: (a–b) treatment modality — LINAC‐based STAR (a) versus CyberKnife (b), (c–d) baseline left ventricular ejection fraction (LVEF) ≤ median (c) versus > median (d), (e–f) patient age ≤ median (e) versus > median (f), and (g–h) underlying cardiomyopathy — ischemic (ICM) (g) versus non‐ischemic (NICM) (h).

Subgroup analyses provided additional insights into factors influencing outcomes. Comparisons between LINAC‐ and CyberKnife‐based treatments revealed similar mortality rates at 12 months (35%, 95% CI: 27%–38% for LINAC vs. 29%, 95% CI: 20%–39% for CyberKnife), with minimal differences in acute toxicity (8%, 95% CI: 4%–13% for LINAC vs. 6%, 95% CI: 1%–12% for CyberKnife). Age‐stratified analysis showed slightly lower mortality at 6 months for patients younger than the median age (14%, 95% CI: 7%–21%) compared to older patients (19%, 95% CI: 11%–28%). Similarly, patients with LVEF above the median had marginally lower mortality at 6 months (13%, 95% CI: 7%–19%) compared to those with LVEF below the median (20%, 95% CI: 13%–27%). Regarding cardiomyopathy types, mortality at 6 months was similar for patients with NICM (16%, 95% CI: 9%–22%) compared to ICM (14%, 95% CI: 7%–21%), while VT burden reduction was higher for NICM group (99% for NICM vs. 59% for ICM). In contrast, VT burden reduction at 6 months demonstrated substantial variation across subgroups. For example, NICM patients showed markedly higher VT reduction (99%) compared to ICM patients (59%), while patients with higher LVEF or younger age also showed greater reductions. These subgroup findings are visualized in Figure 3, with the full summary provided in Table 4. Additional forest plots for mortality and acute toxicity subgroup analyses are included in the Supplement (Figures S1–S3). These subgroup findings should be interpreted as exploratory and hypothesis‐generating, as they are based on study‐level aggregates rather than patient‐level data.

**TABLE 4:** Summary of pooled‐analysis results for STAR in VT: Outcomes include pooled estimates for mortality at 6 and 12 months, reduction in VT burden at 6 months, and grade 3+ adverse events within 90 days. Subgroup analyses evaluate variations by treatment modality (LINAC vs. CyberKnife), LVEF (≤ median vs. > median), patient age (≤ median vs. > median), and cardiomyopathy type (ICM vs. NICM). Results are presented as pooled effect estimates with 95% confidence intervals (CI), Cochran's Q statistics, and heterogeneity (*I* ^2^).

Outcome	Metric	Overall	LINAC vs CyberKnife	LVEF (≤ Median vs > Median)	Age (≤ Median vs > Median)	Cardiomyopathy (ICM vs NICM)
Deaths at 6 months	Pooled Effect	0.16 (0.11, 0.20)	0.16 (0.11,0.22) vs 0.12 (0.02,0.21)	0.20 (0.13,0.27) vs 0.13 (0.07,0.19)	0.14 (0.09,0. 19) vs 0.19 (0.11,0.28)	0.14 (0.07,0.21) vs 0.16 (0.09,0.22)
	Cochran's Q	18.6 (*p* = 0.67)	17.7 (*p* = 0.54) vs 0.16 (*p* = 0.92)	6.44 (*p* = 0.89) vs 9.96 (*p* = 0.35)	10.72 (*p* = 0.55) vs 6.84 (*p* = 0.65)	3.3 (p = 0.86) vs 11.6 (p = 0.31)
	*I* ^2^	0.00%	0.00% vs 0.00%	0.00% vs 9.6%	0.00% vs 0.00%	0.0% vs 13.6%
Deaths at 12 months	Pooled Effect	0.33 (0.27, 0.38)	0.35 (0.26,0.42) vs 0.29 (0.20,0.39)	0.35 (0.26,0.44) vs 0.31 (0.23,0.38)	0.31 (0.24,0.37) vs 0.38 (0.27,0.50)	0.34 (0.26,0.42) vs 0.30 (0.21,0.40)
	Cochran's Q	16.47 (*p* = 0.63)	6.94 (*p* = 0.96) vs 8.71 (*p* = 0.03)	4.54 (*p* = 0.92) vs 10.95 (*p* = 0.18)	13.52 (*p* = 0.26) vs 1.79 (*p* = 0.97)	8.29 (p = 0.41) vs 6.93 (p = 0.44)
	*I* ^2^	0.00%	0.00% vs 65.57%	0.00% vs 29.8%	18.64% vs 0.00%	3.48% vs 0.00%
Grade 3+ Adverse Events within 90 days	Pooled Effect	0.07 (0.04, 0.10)	0.07 (0.03,0.11) vs 0.06 (0.01,0.12)	0.07 (0.03,0.12) vs 0.06 (0.02,0.10)	0.06 (0.01,0.12) vs 0.07 (0.03,0.11)	0.07 (0.02,0.11) vs 0.07 (0.02,0.11)
	Cochran's Q	7.62 (*p* = 0.99)	5.51 (*p* = 0.99) vs 2.07 (*p* = 0.56)	2.33 (*p* = 0.99) vs 5.18 (*p* = 0.88)	1.35 (*p* = 0.99) vs 6.23 (*p* = 0.93)	3.36 (p = 0.91) vs 3.32 (p = 0.98)
	*I* ^2^	0.00%	0.00% vs 0.00%	0.00% vs 0.00%	0.00% vs 0.00%	0.00% vs 0.00%
VT events reduction at 6 months	Pooled Effect	0.75 (0.73, 0.77)	0.98 (0.97,1.00) vs 0.15 (0.02,0.28)	0.69 (0.66,0.72) vs 0.89 (0.79,0.99)	0.77 (0.74,0.80) vs 0.83 (0.73,0.93)	0.59 (0.54,0.64) vs 0.99 (0.97,1.01)
	Cochran's Q	1328.4 (*p* < 0.05)	62.24 (*p* < 0.05) vs 26.42 (*p* < 0.05)	742.60 (*p* < 0.05) vs 5.11 (*p* = 0.40)	760.51 (*p* < 0.05) vs 7.38 (*p* = 0.29)	405.52 (p < 0.05) vs 13.38 (p = 0.06)
	*I* ^2^	98.80%	77.51% vs 96.21%	98.65% vs 2.12%	98.82% vs 18.68%	98.52% vs 47.69%

## 4 DISCUSSION

This synthesis and pooled analysis provides an updated overview of STAR for refractory VT, integrating findings across preclinical reports, case series, and prospective clinical trials. The data reinforce that STAR is primarily utilized in a high‐risk, heavily pretreated patient population—many of whom have failed conventional therapies such as CA and antiarrhythmic drugs. Despite this clinical complexity, early and intermediate outcomes appear promising, particularly with respect to sustained reductions in VT burden and low rates of acute severe toxicity. However, variability in follow‐up intervals, reporting standards, and endpoint definitions limits direct comparability across studies, underscoring the need for harmonized prospective protocols. Collectively, the available literature suggests that STAR is feasible and can provide meaningful short‐term VT reduction with acceptable early toxicity in selected patients. However, important uncertainties remain regarding optimal patient selection, target definition, motion‐management strategy, dose prescription, and long‐term safety, which limits immediate standardization across centers.

Subgroup analyses provided exploratory insights into factors influencing outcomes. Younger patients and those with higher LVEF consistently demonstrated better survival and VT reduction rates. As compared to patients with ICM, NICM patients experienced superior VT burden reduction (99% vs. 59%) and reduced mortality at 12 months (34% vs 30%). These findings suggest that STAR outcomes may vary based on patient‐specific characteristics; however, they should be considered hypothesis‐generating given the study‐level nature of the analysis and potential ecological bias. They nonetheless highlight the potential value of tailored treatment approaches and stratified clinical trial designs. Future studies incorporating individual patient data (IPD) will be necessary to validate these associations and identify true predictors of response.

The high heterogeneity observed in VT burden reduction outcomes (*I* ^2^ = 98.80%) highlights a critical need for standardized reporting and consistent methodologies. Definitions of VT burden varied across studies, with some quantifying episodes per unit time and others measuring total VT events. Additionally, while our pooled analysis standardized the evaluation at a 6‐month timepoint, the definitions of pre‐ and posttreatment intervals and blanking periods were not uniform across studies. Some studies applied a 6‐week blanking period, defined as an initial posttreatment interval during which arrhythmic events are not considered treatment failures due to transient electrophysiologic effects, while others did not mention such adjustments. Moreover, VT burden metrics varied, including sustained VT, all VT/VF events, or ICD interventions. These inconsistencies highlight the need for standardized definitions and outcome intervals in future STAR reporting. Such variability complicates comparisons and pooled analyses, emphasizing the need for standardized reporting of VT burden, follow‐up intervals, ICD programming parameters, and outcome definitions in future STAR studies. In addition, the predominance of relatively small, observational studies may introduce small‐study effects, where studies with favorable outcomes are more likely to be reported, potentially inflating pooled estimates of treatment efficacy. Furthermore, individual studies sometimes contributed disproportionately to pooled estimates. For example, one study (Haskova et al.^101^) reported 17 of 36 deaths at 12 months but did not provide short‐term outcomes, potentially skewing the 12‐month mortality estimate. These imbalances further underscore the importance of reporting outcomes at standardized time intervals to improve cross‐study comparability.

Subgroup analyses suggested that younger patients, those with NICM, and higher LVEF may be associated with more favorable outcomes. Future clinical trials should stratify patients based on these factors and consider incorporating interim analyses or predefined endpoints, such as VT‐free survival, to evaluate efficacy or safety. Trials may also include provisions for early conclusion if predefined thresholds for success or excessive adverse events are met, ensuring patient safety and resource optimization. This approach would not only improve trial efficiency but also minimize risk for high‐risk patients. The results also highlight the need to explore differential outcomes between LINAC‐ and CyberKnife‐based treatments, particularly in terms of toxicity profiles and cost‐effectiveness.

Recently, the first head‐to‐head comparison between STAR and repeat CA reported 3‐year outcomes in 43 patients treated at a high‐volume center.^106^ Patients undergoing STAR experienced markedly fewer serious adverse events (9% vs 38%) and a longer time to toxicity (10 months vs 6 days) compared with CA, while achieving comparable ventricular‐tachycardia control and overall survival (28.2 vs 12.2 months, *P* = .91). Although limited by retrospective design and small sample size, this study provides the first long‐term evidence suggesting that STAR may offer similar efficacy to CA with improved safety in medically fragile populations. These findings further justify ongoing prospective trials directly comparing non‐invasive STAR with repeat invasive ablation.

Accurate motion management remains a cornerstone of STAR. LINAC‐based systems predominantly rely on 4DCT and ITV expansions, while CyberKnife employs fiducial tracking to accommodate respiratory and cardiac motion. Although CyberKnife offers sub‐millimeter precision, its treatment times are significantly longer compared to LINACs (typically on the order of ∼60–120 min vs. ∼20–40 min), posing logistical challenges in a clinical setting. The variability in PTV margins across studies (ranging from 1 to 8 mm isotropic expansions) further underscores the lack of standardization in STAR planning. Additionally, reported dose prescriptions varied in both isodose normalization (e.g., 25 Gy to the 80% vs. 100% isodose line) and motion‐encompassing strategies (e.g., gating, tracking, ITV), which may have implications for BED delivery to arrhythmogenic targets. The feasibility of higher‐dose regimens, as explored in emerging dose‐escalation trials (e.g., DEFT‐STAR), underscores the need to define optimal dose thresholds for efficacy while minimizing toxicity. Addressing these inconsistencies is critical for optimizing treatment precision and minimizing radiation dose to surrounding organs.

From a clinical medical physics perspective, STAR presents unique challenges related to treatment delivery, motion management, dosimetry and quality assurance that must be carefully addressed for safe and reproducible implementation. Consistency in motion‐management strategies, image‐guidance protocols, and dose‐calculation algorithms remains essential for reproducibility across institutions. Discrepancies in PTV margins, dose‐grid resolution, and image‐registration techniques, and treatment delivery parameters (e.g., beam arrangement, tracking or gating strategies) can significantly affect delivered dose to arrhythmogenic targets and adjacent structures. These dosimetric variations may directly influence the biologically effective dose delivered to arrhythmogenic substrates and contribute to variability in treatment response. For example, motion interplay effects in hypofractionated single‐fraction STAR (e.g., 25 Gy) may lead to underdosage of the arrhythmogenic substrate if cardiac and respiratory motion are not adequately accounted for, particularly when using small PTV margins (1–3 mm). Similarly, dose calculation uncertainties arising from heterogeneous thoracic anatomy and the limited temporal resolution of 4DCT may introduce systematic deviations in delivered dose. These factors highlight the importance of patient‐specific motion assessment and robust planning strategies in STAR.″

Establishing standardized QA frameworks—potentially modeled after AAPM TG‐142^107^, TG‐203^108^, and TG‐218^109^—will ensure dosimetric accuracy and patient safety as STAR moves toward routine clinical adoption. Future STAR‐specific QA guidelines may also be warranted, particularly as particle therapy and MRI‐guided systems enter broader clinical use. These should address end‐to‐end testing, including the use of dynamic phantoms to simulate combined cardiac and respiratory motion, motion robustness validation, defined as the ability of the treatment to maintain target coverage despite cardiac and respiratory motion, and system latency considerations in motion‐managed or tracked delivery. These considerations highlight the importance of robust motion‐management strategies and delivery accuracy in ensuring consistent dose delivery to dynamic cardiac targets.

In addition, multidisciplinary coordination between medical physics, electrophysiology, and radiology teams is essential to ensure accurate integration of imaging, mapping data, and treatment delivery within the STAR workflow. These considerations highlight the central role of clinical medical physics in ensuring accurate, reproducible, and safe implementation of STAR across institutions.

Particle therapies, such as protons and carbon ions, represent an emerging frontier in STAR, particularly for younger patients or those with complex anatomies. The ability to leverage the Bragg peak for precise dose deposition makes these modalities uniquely suited for cases involving critical adjacent structures, such as the esophagus and lungs. Preclinical studies have demonstrated the feasibility of particle therapy for arrhythmia ablation^35^, ^36^; however, clinical data remain sparse. Dusi et al.^49^ and Amino et al.^77^ successfully demonstrated the first‐in‐human use of protons and carbons to treat VT and demonstrated a reduction in VT events post STAR.

Lee et al.^53^ treated an 11‐year‐old pediatric patient for VT with photons. While the patient was in good condition at the 3‐month follow‐up visit, long‐term follow‐up data are unavailable, and this patient might have benefited from proton therapy, given its dosimetric advantages. Shah et al.^110^ demonstrated significant reductions in OAR doses for retrospectively planned patients treated with proton therapy compared with corresponding photon plans. Despite these promising developments, challenges such as range uncertainties, motion management, and the high costs of particle therapy must be addressed to facilitate its broader adoption.

Grade 3+ adverse events were rare (7%, 95% CI: 4%–10%) but underscore the importance of meticulous planning to avoid significant complications. The reported cases of esophagitis and pneumonitis, particularly in patients with posterior substrates, emphasize the need for advanced planning techniques and possibly proton therapy to spare critical structures. Moreover, attention must be paid to the radiation dose delivered to ICDs, as inappropriate shocks or device malfunctions remain a concern. Guidelines such as AAPM TG‐203^108^ offer practical recommendations for managing these challenges during treatment.

Cases requiring repeated STAR treatments illustrate the challenges of achieving complete arrhythmic suppression in patients with extensive or complex arrhythmogenic substrates. Improved target identification, supported by EAM, ECGI, and advanced imaging modalities, can mitigate the need for retreatments. Artificial intelligence (AI)‐based automated and semi‐automated models have shown promise in automating substrate delineation, reducing inter‐observer variability, and identifying non‐responders earlier in the treatment course.^111^, ^112^, ^113^, ^114^ Integration of physics‐based dose‐response modeling with AI‐driven substrate delineation could further individualize STAR planning and enable biologically adaptive treatment strategies.

The radiobiological efficacy of STAR is underpinned by its ability to induce fibrosis and modulate myocardial conduction pathways, disrupting arrhythmogenic circuits. However, the biological mechanisms remain incompletely understood. Preclinical studies have highlighted changes in gap junction remodeling (e.g., connexin‐43 expression), conduction slowing, and fibrosis as key contributors to arrhythmia suppression.^26^, ^27^ Long‐term consequences of radiation, including vascular damage and inflammatory responses, require further investigation, particularly in younger patients who may face increased risks of late toxicities.

The overwhelming majority of STAR studies have been conducted in North America, Europe, and East Asia, with limited representation from South America, Africa, the Middle East, and the Indian subcontinent. This geographic disparity reflects broader inequities in access to advanced radiation therapy technologies. Efforts must be made to globalize radiation therapy, making STAR accessible to all patients who need it. This includes reducing financial and logistical barriers to acquiring treatment infrastructure and fostering international collaboration to ensure equitable access.

This study has several limitations. First, although the pooled analyses were conducted using established meta‐analytic techniques, the review itself was not prospectively registered (e.g., on PROSPERO), and a formal risk‐of‐bias assessment was not performed. This is primarily because the review was designed as a narrative synthesis with exploratory pooled analysis rather than a formal systematic review. We focused on hypothesis generation using study‐level data, without access to IPD, acknowledging the variability in study designs, reporting quality, and follow‐up durations. Accordingly, the pooled estimates should be interpreted as exploratory rather than definitive. Second, the use of a single bibliographic database (PubMed) may limit search comprehensiveness, though we mitigated this by manually reviewing reference lists and citations of included studies. Third, publication bias, defined as the preferential reporting of studies with favorable or positive outcomes, cannot be excluded, and high heterogeneity was observed—particularly in VT burden reduction outcomes—reflecting inconsistency in endpoint definitions, blanking periods, and reporting standards. Fourth, unmeasured confounding may have influenced the results. Variables such as antiarrhythmic drug use, NYHA status, comorbidities and ICD programming were inconsistently reported, limiting adjustment in pooled analyses. Fifth, although only a small number of studies were excluded based on language, this may still introduce minor selection bias and underrepresentation of certain geographic regions. Sixth, female patients were underrepresented across included studies (approximately 89% of patients were male), limiting sex‐specific insights. Lastly, long‐term outcomes remain sparsely reported, and the lack of randomized controlled trials limits causal inference. Future prospective, standardized trials—ideally with diverse patient populations and harmonized data collection—are needed to further define the role of STAR in VT management. Standardization of STAR protocols and reporting practices is critical to advancing this field. Collaborative efforts are needed to develop robust frameworks for patient selection, target delineation, and outcome reporting. Future research should focus on:

1. Expanding STAR to earlier‐stage VT patients, including those without prior CA failure.
2. Exploring particle therapy, particularly protons, for cases requiring enhanced OAR sparing.
3. Investigating advanced imaging integration (e.g., multimodality fusion of ECGI or EAM with CT/MRI) and motion management techniques (e.g., gating, tracking, or 4DCT‐based approaches) to improve target definition and precision.
4. Addressing geographic disparities by fostering international collaborations and improving access to radiation therapy technologies in underserved regions.

The findings of this review and pooled analysis underscore the transformative potential of STAR in VT management, while highlighting opportunities to refine and expand its application. Addressing the outlined challenges will be critical to maximizing STAR's clinical impact and ensuring equitable access to this life‐saving technology. As clinical and technical evidence converge, multidisciplinary collaboration between electrophysiology and medical physics will be critical to standardize planning, QA, and follow‐up metrics for STAR.

## 5 Conclusion

In conclusion, STAR is poised to reshape the landscape of VT management by offering a noninvasive, image‐guided alternative for patients ineligible for or refractory to CA. This review underscores its capacity to meaningfully reduce arrhythmic burden with acceptable acute toxicity, while also highlighting the wide variability in patient selection, planning methods, and outcome reporting across institutions. As STAR evolves from a salvage therapy to a more structured treatment paradigm, multidisciplinary collaboration will be essential—linking clinical cardiology and electrophysiology with medical physics, imaging, and radiotherapy. Continued prospective studies, technical standardization, and broader global access will be critical to realizing the full therapeutic potential of STAR in ventricular arrhythmia care.

## AUTHOR CONTRIBUTIONS

Keyur D. Shah: Conceptualization; Methodology; Data curation; Software; Formal analysis; Investigation; Visualization; Writing—original draft. Chih‐Wei Chang: Writing review and editing. Sibo Tian: Writing—review and editing. Pretesh Patel: Writing—review and editing. Richard Qiu: Writing—review and editing. Shadab Momin: Writing—review and editing. Justin Roper: Writing—review and editing. Jun Zhou: Writing—review and editing. Zhen Tian: Writing—review and editing. Xiaofeng Yang: Conceptualization; Supervision; Funding acquisition; Project administration; Writing—review and editing.

## CONFLICT OF INTEREST STATEMENT

The authors declare no conflicts of interest.

## FUNDING INFORMATION

This research is supported in part by the National Institutes of Health under Award Number R01CA272991, R01EB032680, R37CA272755 and U54CA274513.

## Supporting information



Supporting Information

Supporting Information

Supporting Information

## References

[1] Koplan BA , Stevenson WG . Ventricular tachycardia and sudden cardiac death. Mayo Clin Proc 2009;84(3):289–297. 19252119 10.4065/84.3.289 PMC2664600

[2] Harris P , Lysitsas D . Ventricular arrhythmias and sudden cardiac death. BJA Education 2016;16(7):221–229.

[3] Soejima K , Stevenson WG , Sapp JL , Selwyn AP , Couper G , Epstein LM . Endocardial and epicardial radiofrequency ablation of ventricular tachycardia associated with dilated cardiomyopathy. J Am Coll Cardiol. 2004;43(10):1834–1842. 15145109 10.1016/j.jacc.2004.01.029

[4] Zeppenfeld K . Ventricular tachycardia ablation in nonischemic cardiomyopathy. JACC 2018;4(9):1123–1140. 30236385 10.1016/j.jacep.2018.06.014

[5] Anter E , Tschabrunn CM , Buxton AE , Josephson ME . High‐resolution mapping of postinfarction reentrant ventricular tachycardia: Electrophysiological characterization of the circuit. Circulation 2016;134(4):314–327. 27440005 10.1161/CIRCULATIONAHA.116.021955 PMC5072375

[6] Ciaccio EJ , Coromilas J , Wit AL , Peters NS , Garan H . Formation of functional conduction block during the onset of reentrant ventricular tachycardia. Circ Arrhythm Electrophysiol 2016;9(12):e004462. 27879278 10.1161/CIRCEP.116.004462

[7] Ibrahim R , Sroubek J , Nakhla S , Lee JZ . Trends and disparities in ventricular tachycardia mortality in the United States. Cardiovasc electrophysiol 2023;34(2):465–467. 10.1111/jce.15812 PMC10107254 36640434

[8] Santangeli P , Muser D , Maeda S , et al. Comparative effectiveness of antiarrhythmic drugs and catheter ablation for the prevention of recurrent ventricular tachycardia in patients with implantable cardioverter‐defibrillators: A systematic review and meta‐analysis of randomized controlled trials. Heart Rhythm 2016; 13(7): 1552–1559. 26961297 10.1016/j.hrthm.2016.03.004

[9] Mason JW . A comparison of seven antiarrhythmic drugs in patients with ventricular tachyarrhythmias. N Engl J Med 1993;329(7):452–458. 8332150 10.1056/NEJM199308123290702

[10] Maron BJ , Estes NAM , Rowin EJ , Maron MS , Reynolds MR . Development of the implantable cardioverter‐defibrillator. J Am Coll Cardiol 2023;82(4):353–373. 37468191 10.1016/j.jacc.2023.04.056

[11] Thomas SA , Friedmann E , Kao C‐W , et al. Quality of life and psychological status of patients with implantable cardioverter defibrillators. Am J Crit Care 2006; 15(4): 389–398. 16823016

[12] Stevenson WG , Soejima K . Catheter ablation for ventricular tachycardia. Circulation 2007;115(21):2750–2760. 17533195 10.1161/CIRCULATIONAHA.106.655720

[13] Palaniswamy C , Kolte D , Harikrishnan P , et al. Catheter ablation of postinfarction ventricular tachycardia: Ten‐year trends in utilization, in‐hospital complications, and in‐hospital mortality in the United States. Heart Rhythm 2014;11(11):2056–2063. 25016150 10.1016/j.hrthm.2014.07.012

[14] Santangeli P , Frankel DS , Tung R , et al. Early mortality after catheter ablation of ventricular tachycardia in patients with structural heart disease. J Am Coll Cardiol. 2017;69(17):2105–2115. 28449770 10.1016/j.jacc.2017.02.044

[15] Jadczyk T , Miszczyk M , Kurzelowski R , et al. Stereotactic radioablation for treatment of ventricular tachycardia, In: Jadczyk T , Caluori G , Loewe A and Golba KS eds. Innovative Treatment Strategies for Clinical Electrophysiology Springer Nature Singapore; 2022: 1–27.

[16] Miyashita T , Miyazawa I , Kawaguchi T , et al. A case of primary cardiac B cell lymphoma associated with ventricular tachycardia, successfully treated with systemic chemotherapy and radiotherapy: a long‐term survival case. Jpn Circ J 2000;64(2):135–138. 10716528 10.1253/jcj.64.135

[17] Tanaka Y , Yamabe H , Yamasaki H , et al. A case of reversible ventricular tachycardia and complete atrioventricular block associated with primary cardiac B‐cell lymphoma. Pacing Clin Electrophysiol 2009;32(6):816–819. 19545348 10.1111/j.1540-8159.2009.02372.x

[18] Loo BWJ , Soltys SG , Wang L , et al. Stereotactic ablative radiotherapy for the treatment of refractory cardiac ventricular arrhythmia. Circ Arrhythm Electrophysiol 2015;8(3):748–750. 26082532 10.1161/CIRCEP.115.002765

[19] Cuculich PS , Schill MR , Kashani R , et al. Noninvasive cardiac radiation for ablation of ventricular tachycardia. N Engl J Med 2017;377(24):2325–2336. 29236642 10.1056/NEJMoa1613773 PMC5764179

[20] Viani GA , Gouveia AG , Pavoni JF , et al. A meta‐analysis of the efficacy and safety of stereotactic arrhythmia radioablation (STAR) in patients with refractory ventricular tachycardia. Clin Oncol (R Coll Radiol) 2023;35(9):611–620. 37365062 10.1016/j.clon.2023.04.004

[21] Benali K , Zei PC , Lloyd M , et al. One‐year mortality and causes of death after stereotactic radiation therapy for refractory ventricular arrhythmias: A systematic review and pooled analysis. Trends Cardiovasc Med 2024;34(7):488–496. 38191005 10.1016/j.tcm.2023.12.008

[22] Miszczyk M , Hoeksema WF , Kuna K , et al. Stereotactic arrhythmia radioablation (STAR)‐a systematic review and meta‐analysis of prospective trials on behalf of the STOPSTORM.eu consortium. Heart Rhythm 2025;22(1):80–89. doi:10:1016/j.hrthm.2024.07.029 39032525 10.1016/j.hrthm.2024.07.029

[23] Gupta A , Sattar Z , Chaaban N , et al. Stereotactic cardiac radiotherapy for refractory ventricular tachycardia in structural heart disease patients: a systematic review. Europace 2024;27(1):euae305. 39716963 10.1093/europace/euae305 PMC11780863

[24] Greiner A , Grajewski L , Römer M , Pietschmann K , Wurschi G . Technical aspects of SBRT for therapy‐refractory ventricular tachycardia: a systematic review for radiation oncologists. Radiat Oncol 2025;20(1):136. 40883833 10.1186/s13014-025-02704-w PMC12395866

[25] Page MJ , McKenzie JE , Bossuyt PM , et al. The PRISMA 2020 statement: an updated guideline for reporting systematic reviews. BMJ 2021;372:n71. 33782057 10.1136/bmj.n71 PMC8005924

[26] Amino M , Yoshioka K , Furusawa Y , et al. Inducibility of ventricular arrhythmia 1 year following treatment with heavy ion irradiation in dogs with myocardial infarction. Pacing Clin Electrophysiol 2017;40(4):379–390. 28158934 10.1111/pace.13031

[27] Amino M , Yamazaki M , Yoshioka K , et al. Heavy ion irradiation reduces vulnerability to atrial tachyarrhythmias ‐ gap junction and sympathetic neural remodeling. Circ J. 2023;87(7):1016–1026. 36476829 10.1253/circj.CJ-22-0527

[28] Lehmann HI , Richter D , Prokesch H , et al. Atrioventricular node ablation in Langendorff‐perfused porcine hearts using carbon ion particle therapy: methods and an in vivo feasibility investigation for catheter‐free ablation of cardiac arrhythmias. Circ Arrhythm Electrophysiol 2015;8(2):429–438. 25609687 10.1161/CIRCEP.114.002436

[29] Lehmann HI , Deisher AJ , Takami M , et al. External arrhythmia ablation using photon beams: Ablation of the atrioventricular junction in an intact animal model. Circ Arrhythm Electrophysiol 2017;10(4):e004304. 28408649 10.1161/CIRCEP.116.004304

[30] Zei PC , Wong D , Gardner E , Fogarty T , Maguire P . Safety and efficacy of stereotactic radioablation targeting pulmonary vein tissues in an experimental model. Heart Rhythm 2018;15(9):1420–1427. 29678783 10.1016/j.hrthm.2018.04.015

[31] Hohmann S , Deisher AJ , Suzuki A , et al. Left ventricular function after noninvasive cardiac ablation using proton beam therapy in a porcine model. Heart Rhythm 2019;16(11):1710–1719. 31004779 10.1016/j.hrthm.2019.04.030

[32] Hohmann S , Deisher AJ , Konishi H , et al. Catheter‐free ablation of infarct scar through proton beam therapy: Tissue effects in a porcine model. Heart Rhythm 2020;17(12):2190–2199. 32673796 10.1016/j.hrthm.2020.07.011

[33] Kancharla K , Olson A , Salavatian S , et al. Ventricular arrhythmia inducibility in porcine infarct model after stereotactic body radiation therapy. Heart Rhythm 2024;21(7):1154–1160. 38395245 10.1016/j.hrthm.2024.02.037

[34] Kim BH , Jung JW , Han D , Cha M‐J , Chang JH . One‐week dynamic changes in cardiac proteomes after cardiac radioablation in experimental rat model. Front Cardiovasc Med 2022;9:898222. 35837601 10.3389/fcvm.2022.898222 PMC9273889

[35] Takami M , Hara T , Okimoto T , et al. Electrophysiological and pathological impact of medium‐dose external carbon ion and proton beam radiation on the left ventricle in an animal model. J Am Heart Assoc 2021;10(7):e019687. 33759547 10.1161/JAHA.120.019687 PMC8174335

[36] Imamura K , Deisher AJ , Dickow J , et al. Early impact of proton beam therapy on electrophysiological characteristics in a porcine model. Circ Arrhythm Electrophysiol 2023;16(6):e011179. 37183678 10.1161/CIRCEP.122.011179

[37] Vaskovskii VA , Taimasova IA , Artyukhina EA , Golanov AV , Revishvili AS . Development of noninvasive technology of stereotaxic radioablation using linear accelerators for the treatment of life‐threatening ventricular tachycardias in experiment. Bull Exp Biol Med 2022;172(5):612–616. 35352259 10.1007/s10517-022-05445-y

[38] Amino M , Shimokawa T , Wakizaka H , et al. Noninvasive imaging reveals cardiac sympathetic reinnervation in pathologic hearts and suppressed norepinephrine transporter function in healthy hearts after carbon ion irradiation in rabbits. Heart Rhythm 2025;22(12):3277‐3288. doi:10.1016/j.hrthm.2025.06.001 40484161

[39] Jumeau R , Ozsahin M , Schwitter J , et al. Rescue procedure for an electrical storm using robotic non‐invasive cardiac radio‐ablation. Radiother Oncol 2018;128(2):189–191. 29753550 10.1016/j.radonc.2018.04.025

[40] Haskova J , Peichl P , Pirk J , Cvek J , Neuwirth R , Kautzner J . Stereotactic radiosurgery as a treatment for recurrent ventricular tachycardia associated with cardiac fibroma. HeartRhythm Case Rep 2019;5(1):44–47. 30693205 10.1016/j.hrcr.2018.10.007 PMC6342609

[41] Haskova J , Peichl P , Šramko M , et al. Case report: Repeated stereotactic radiotherapy of recurrent ventricular tachycardia: Reasons, feasibility, and safety. Front Cardiovasc Med 2022;9:845382. 35425817 10.3389/fcvm.2022.845382 PMC9004321

[42] Bhaskaran A , Downar E , Chauhan VS , et al. Electroanatomical mapping‐guided stereotactic radiotherapy for right ventricular tachycardia storm. HeartRhythm Case Rep 2019;5(12):590–592. 31890583 10.1016/j.hrcr.2019.09.007 PMC6926178

[43] Zeng L‐J , Huang L‐H , Tan H , et al. Stereotactic body radiation therapy for refractory ventricular tachycardia secondary to cardiac lipoma: A case report. Pacing Clin Electrophysiol 2019;42(9):1276–1279. 31116434 10.1111/pace.13731

[44] Martí‐Almor J , Jiménez‐López J , Rodríguez de Dios N , Tizón H , Vallés E , Algara M . Noninvasive ablation of ventricular tachycardia with stereotactic radiotherapy in a patient with arrhythmogenic right ventricular cardiomyopathy. Rev Esp Cardiol (Engl Ed) 2020;73(1):97–99. 31375392 10.1016/j.rec.2019.06.004

[45] Narducci ML , Cellini F , Placidi L , et al. Case report: a case report of stereotactic ventricular arrhythmia radioablation (STAR) on large cardiac target volume by highly personalized inter‐ and intra‐fractional image guidance. Front Cardiovasc Med 2020;7:565471. 33330640 10.3389/fcvm.2020.565471 PMC7719630

[46] Mayinger M , Kovacs B , Tanadini‐Lang S , et al. First magnetic resonance imaging‐guided cardiac radioablation of sustained ventricular tachycardia. Radiother Oncol 2020;152:203–207. 32067819 10.1016/j.radonc.2020.01.008

[47] Krug D , Blanck O , Demming T , et al. Stereotactic body radiotherapy for ventricular tachycardia (cardiac radiosurgery) : First‐in‐patient treatment in Germany. Strahlenther Onkol 2020;196(1):23–30. 31673718 10.1007/s00066-019-01530-w

[48] Park JS , Choi Y . Stereotactic cardiac radiation to control ventricular tachycardia and fibrillation storm in a patient with apical hypertrophic cardiomyopathy at burnout stage: case report. J Korean Med Sci 2020;35(27):e200. 32657082 10.3346/jkms.2020.35.e200 PMC7358068

[49] Dusi V , Vitolo V , Frigerio L , et al. First‐in‐man case of non‐invasive proton radiotherapy for the treatment of refractory ventricular tachycardia in advanced heart failure. Eur J Heart Fail 2021;23(1):195–196. 33179329 10.1002/ejhf.2056

[50] Peichl P , Sramko M , Cvek J , Kautzner J . A case report of successful elimination of recurrent ventricular tachycardia by repeated stereotactic radiotherapy: the importance of accurate target volume delineation. Eur Heart J Case Rep 2021;5(2):ytaa516. 33598611 10.1093/ehjcr/ytaa516 PMC7873794

[51] Amino M , Kabuki S , Kunieda E , Yagishita A , Ikari Y , Yoshioka K . Analysis of depolarization abnormality and autonomic nerve function after stereotactic body radiation therapy for ventricular tachycardia in a patient with old myocardial infarction. HeartRhythm Case Rep 2021;7(5):306–311. 34026521 10.1016/j.hrcr.2021.01.023 PMC8134781

[52] Quick S , Christoph M , Polster D , Ibrahim K , Schöpe M , Klautke G . Immediate response to electroanatomical mapping‐guided stereotactic ablative radiotherapy for ventricular tachycardia. Radiat Res 2021;195(6):596–599. 33826732 10.1667/RADE-21-00011.1

[53] Lee Y , Yoon HI , Kim J‐S , Kim A‐Y , Tsevendee S , Uhm J‐S . Incessant ventricular tachycardia treated with cardiac radioablation in an 11‐year‐old boy with dilated cardiomyopathy. HeartRhythm Case Rep 2021;7(3):186–190. 33786318 10.1016/j.hrcr.2020.12.009 PMC7987900

[54] Kautzner J , Jedlickova K , Sramko M , et al. Radiation‐induced changes in ventricular myocardium after stereotactic body radiotherapy for recurrent ventricular tachycardia. JACC Clin Electrophysiol 2021;7(12):1487–1492. 34600851 10.1016/j.jacep.2021.07.012

[55] Thosani A , Trombetta M , Shaw G , Oh S , Sohn J , Liu E . Stereotactic arrhythmia radioablation for intramural basal septal ventricular tachycardia originating near the His bundle. HeartRhythm Case Rep 2021;7(4):246–250. 34026506 10.1016/j.hrcr.2021.01.012 PMC8129041

[56] Aras D , Ozturk HF , Ozdemir E , et al. Use of stereotactic radioablation therapy as a bailout therapy for refractory ventricular tachycardia in a patient with a no‐entry left ventricle. J Innov Card Rhythm Manag 2021;12(9):4671–4675. 34595050 10.19102/icrm.2021.120902 PMC8476093

[57] Li J , Chen Q , Li G , et al. Stereotactic arrhythmia radiotherapy: a case study of real‐time image‐guided noninvasive treatment for ventricular tachycardia. Quant Imaging Med Surg 2022;12(4):2607–2615. 35371930 10.21037/qims-21-1025 PMC8923871

[58] Hayase J , Chin R , Kwon M , et al. Surgical ablation after stereotactic body radiation therapy for ventricular arrhythmias. HeartRhythm Case Rep 2022;8(2):73–76. 35242541 10.1016/j.hrcr.2021.10.006 PMC8858747

[59] Levis M , Dusi V , Magnano M , et al. A case report of long‐term successful stereotactic arrhythmia radioablation in a cardiac contractility modulation device carrier with giant left atrium, including a detailed dosimetric analysis. Front Cardiovasc Med 2022;9:934686. 36072883 10.3389/fcvm.2022.934686 PMC9441661

[60] Huang S‐H , Wu Y‐W , Shueng P‐W , et al. Case report: Stereotactic body radiation therapy with 12 Gy for silencing refractory ventricular tachycardia. Front Cardiovasc Med 2022;9:973105. 36407435 10.3389/fcvm.2022.973105 PMC9669661

[61] van der Ree MH , Dieleman EMT , Visser J , et al. Direct clinical effects of cardiac radioablation in the treatment of a patient with therapy‐refractory ventricular tachycardia storm. Adv Radiat Oncol 2022;7(5):100992. 35782880 10.1016/j.adro.2022.100992 PMC9240979

[62] Wutzler A , Tiedke B , Osman M , Mahrous N , Wurm R . Elimination of refractory ventricular tachycardia storm and fibrillation using stereotactic radiotherapy. Clin Case Rep 2023;11(1):e6690. 36694642 10.1002/ccr3.6690 PMC9842778

[63] Bernstein HM , Leon W , Daly ME , et al. Noninvasive stereotactic radiation for refractory ventricular tachycardia after failure of cardiac sympathetic denervation. JACC Case Rep 2022;4(18):1189–1194. 36213875 10.1016/j.jaccas.2022.06.025 PMC9537071

[64] Kurzelowski R , Latusek T , Miszczyk M , et al. Radiosurgery in treatment of ventricular tachycardia ‐ initial experience within the polish SMART‐VT trial. Front Cardiovasc Med 2022;9:874661. 35509272 10.3389/fcvm.2022.874661 PMC9058092

[65] Cybulska M , Sajdok M , Bednarek J , et al. Stereotactic arrhythmia radioablation in recurrent ventricular tachyarrhythmias. Kardiol Pol 2022;80(3):367–369. 35076080 10.33963/KP.a2022.0019

[66] Ninni S , Longere B , Mirabel X . Stereotactic radioablation to treat ventricular tachycardia related to a left ventricular mass. Eur Heart J 2022;43(24):2341. 35452120 10.1093/eurheartj/ehac218

[67] Nasu T , Toba M , Nekomiya N , et al. Successful application of stereotactic body radiation therapy for ventricular tachycardia substrate in a patient with nonischemic cardiomyopathy. Am J Cardiol 2022;184:149–153. 36163052 10.1016/j.amjcard.2022.08.017

[68] Pavone C , Scacciavillani R , Narducci ML , et al. Successful ventricular tachycardia radioablation in a patient with previous chemical pleurodesis: A case report. Front Cardiovasc Med 2022;9:937090. 35924213 10.3389/fcvm.2022.937090 PMC9339650

[69] Cozzi S , Bottoni N , Botti A , et al. The use of cardiac stereotactic radiation therapy (sbrt) to manage ventricular tachycardia: a case report, review of the literature and technical notes. J Pers Med 2022;12(11):1783. 36579492 10.3390/jpm12111783 PMC9694192

[70] Mehrhof F , Bergengruen P , Gerds‐Li J‐H , et al. Cardiac radioablation of incessant ventricular tachycardia in patients with terminal heart failure under permanent left ventricular assist device therapy‐description of two cases. Strahlenther Onkol 2023;199(5):511–519. 36750509 10.1007/s00066-023-02045-1 PMC10133058

[71] Jiwani S , Akhavan D , Reddy M , Noheria A . Cardiac stereotactic radiotherapy for refractory ventricular tachycardia in a patient with wireless left ventricular endocardial stimulation system. HeartRhythm Case Rep 2023;9(11):818–822. 38023677 10.1016/j.hrcr.2023.08.013 PMC10667127

[72] van der Ree MH , Herrera Siklody C , Le Bloa M , et al. Case report: First‐in‐human combined low‐dose whole‐heart irradiation and high‐dose stereotactic arrhythmia radioablation for immunosuppressive refractory cardiac sarcoidosis and ventricular tachycardia. Front Cardiovasc Med 2023;10:1213165. 37547255 10.3389/fcvm.2023.1213165 PMC10401040

[73] Kaestner L , Boda‐Heggemann J , Fanslau H , et al. Electroanatomical mapping after cardiac radioablation for treatment of incessant electrical storm: a case report from the RAVENTA trial. Strahlenther Onkol 2023;199(11):1018–1024. 37698592 10.1007/s00066-023-02136-z PMC10598131

[74] Wijesuriya N , Galante JR , Sisodia C , Whitaker J , Ahmad S , Rinaldi CA . Increase in right ventricular lead pacing threshold following stereotactic ablative therapy for ventricular tachycardia. HeartRhythm Case Rep 2023;9(8):555–559. 37614389 10.1016/j.hrcr.2023.05.011 PMC10444549

[75] Vaskovskii VA , Taimasova IA , Artyukhina EA , et al. Long‐term results of the first clinical application of stereotactic radioablation using a linear electron accelerator for the treatment of ventricular tachycardia. Bull Exp Biol Med 2023;174(5):594–600. 37052858 10.1007/s10517-023-05753-x

[76] Keyt LK , Atwood T , Bruggeman A , et al. Successful noninvasive 12‐lead ECG mapping‐guided radiotherapy of inaccessible ventricular tachycardia substrate due to mechanical valves. JACC Case Rep 2023;15:101870. 37283824 10.1016/j.jaccas.2023.101870 PMC10240275

[77] Amino M , Wakatsuki M , Mori S , et al. Case of successful sympathetic nerve modulation by targeted heavy ion radiotherapy for idiopathic ventricular tachycardia. Ann Noninvasive Electrocardiol 2024;29(6):e70020. 39425937 10.1111/anec.70020 PMC11490255

[78] Kautzner J , Hašková J , Cvek J , Adamíra M , Peichl P . Hypertrophic obstructive cardiomyopathy with recurrent ventricular tachycardias: from catheter ablation and stereotactic radiotherapy to heart transplant‐a case report. Eur Heart J Case Rep 2024;8(8):ytae379. 39144539 10.1093/ehjcr/ytae379 PMC11322737

[79] Kaya YS , Stoks J , Hazelaar C , et al. 3D‐targeted, electrocardiographic imaging‐aided stereotactic radioablation for ventricular tachycardia storm: a case report. Eur Heart J Case Rep 2024;8(10):ytae541. 39678105 10.1093/ehjcr/ytae541 PMC11638725

[80] Trinh K , Kou A . Palliative stereotactic body radiation therapy for the treatment of refractory ventricular tachycardia. Cureus. 2025;17(3):e80001. 40182370 10.7759/cureus.80001 PMC11966081

[81] Cravéreau O , Stefani A , Buchheit I , et al. Novel use of stereotactic ablative radiotherapy for refractory ventricular tachycardia with cardiac metastasis of primary renal cell carcinoma: a case report. Cardiooncology. 2025;11(1):91. 41094681 10.1186/s40959-025-00392-9 PMC12522970

[82] Robinson CG , Samson PP , Moore KMS , et al. Phase I/II Trial of Electrophysiology‐Guided Noninvasive Cardiac Radioablation for Ventricular Tachycardia. Circulation. 2019; 139(3): 313–321. 30586734 10.1161/CIRCULATIONAHA.118.038261 PMC6331281

[83] Chin R , Hayase J , Hu P , et al. Non‐invasive stereotactic body radiation therapy for refractory ventricular arrhythmias: an institutional experience. J Interv Card Electrophysiol 2021;61(3):535–543. 32803639 10.1007/s10840-020-00849-0

[84] Gianni C , Rivera D , Burkhardt JD , et al. Stereotactic arrhythmia radioablation for refractory scar‐related ventricular tachycardia. Heart Rhythm 2020;17(8):1241–1248. 32151737 10.1016/j.hrthm.2020.02.036

[85] Lee J , Bates M , Shepherd E , et al. Cardiac stereotactic ablative radiotherapy for control of refractory ventricular tachycardia: initial UK multicentre experience. Open Heart 2021;8(2):e001770. 34815300 10.1136/openhrt-2021-001770 PMC8611439

[86] Yugo D , Lo L‐W , Wu Y‐H , et al. Case series on stereotactic body radiation therapy in non‐ischemic cardiomyopathy patients with recurrent ventricular tachycardia. Pacing Clin Electrophysiol. 2021;44(6):1085–1093. 33932305 10.1111/pace.14254

[87] Ho G , Atwood TF , Bruggeman AR , et al. Computational ECG mapping and respiratory gating to optimize stereotactic ablative radiotherapy workflow for refractory ventricular tachycardia. Heart Rhythm O2. 2021;2(5):511–520. 34667967 10.1016/j.hroo.2021.09.001 PMC8505208

[88] Carbucicchio C , Andreini D , Piperno G , et al. Stereotactic radioablation for the treatment of ventricular tachycardia: preliminary data and insights from the STRA‐MI‐VT phase Ib/II study. J Interv Card Electrophysiol. 2021;62(2):427–439. 34609691 10.1007/s10840-021-01060-5 PMC8490832

[89] Qian PC , Quadros K , Aguilar M , et al. Substrate modification using stereotactic radioablation to treat refractory ventricular tachycardia in patients with ischemic cardiomyopathy. JACC Clin Electrophysiol. 2022;8(1):49–58. 34364832 10.1016/j.jacep.2021.06.016

[90] Lloyd MS , Wight J , Schneider F , et al. Clinical experience of stereotactic body radiation for refractory ventricular tachycardia in advanced heart failure patients. Heart Rhythm. 2020;17(3):415–422. 31585181 10.1016/j.hrthm.2019.09.028

[91] Wight J , Bigham T , Schwartz A , et al. Long term follow‐up of stereotactic body radiation therapy for refractory ventricular tachycardia in advanced heart failure patients. Front Cardiovasc Med. 2022;9:849113. 35571173 10.3389/fcvm.2022.849113 PMC9098944

[92] Molon G , Giaj‐Levra N , Costa A , et al. Stereotactic ablative radiotherapy in patients with refractory ventricular tachyarrhythmia. Eur Heart J Suppl . 2022;24(Suppl C):C248–C253. 10.1093/eurheartj/suac016 PMC9117912 35602256

[93] Ninni S , Gallot‐Lavallée T , Klein C , et al. Stereotactic radioablation for ventricular tachycardia in the setting of electrical storm. Circ Arrhythm Electrophysiol. 2022;15(9):e010955. 36074658 10.1161/CIRCEP.122.010955

[94] Chang WI , Jo HH , Cha M‐J , et al. Short‐term and long‐term effects of noninvasive cardiac radioablation for ventricular tachycardia: A single‐center case series. Heart Rhythm O2. 2023;4(2):119–126. 36873313 10.1016/j.hroo.2022.11.006 PMC9975004

[95] Aras D , Çetin EHÖ, Ozturk HF , et al. Stereotactic body radioablation therapy as an immediate and early term antiarrhythmic palliative therapeutic choice in patients with refractory ventricular tachycardia. J Interv Card Electrophysiol. 2023;66(1):135–143. 36040658 10.1007/s10840-022-01352-4 PMC9424800

[96] van der Ree MH , Dieleman EMT , Visser J , et al. Non‐invasive stereotactic arrhythmia radiotherapy for ventricular tachycardia: results of the prospective STARNL‐1 trial. Europace. 2023;25(3):1015–1024. 36746553 10.1093/europace/euad020 PMC10062344

[97] Krug D , Zaman A , Eidinger L , et al. Radiosurgery for ventricular tachycardia (RAVENTA): interim analysis of a multicenter multiplatform feasibility trial. Strahlenther Onkol 2023;199(7):621–630. 37285038 10.1007/s00066-023-02091-9 PMC10245341

[98] Herrera Siklody C , Schiappacasse L , Jumeau R , et al. Recurrences of ventricular tachycardia after stereotactic arrhythmia radioablation arise outside the treated volume: analysis of the Swiss cohort. Europace. 2023;25(10):euad268. 37695314 10.1093/europace/euad268 PMC10551232

[99] Miszczyk M , Sajdok M , Bednarek J , et al. Stereotactic management of arrhythmia ‐ radiosurgery in treatment of ventricular tachycardia (SMART‐VT). Results of a prospective safety trial. Radiother Oncol 2023;188:109857. 37597807 10.1016/j.radonc.2023.109857

[100] Amino M , Kabuki S , Kunieda E , et al. Interim report of a Japanese Phase II trial for cardiac stereotactic body radiotherapy in refractory ventricular tachycardia ― focus on target determination ―. Circ Rep 2023;5(3):69–79. 36909137 10.1253/circrep.CR-23-0003 PMC9992511

[101] Hašková J , Wichterle D , Kautzner J , et al. Efficacy and safety of stereotactic radiotherapy in patients with recurrent ventricular tachycardias: the Czech experience. JACC Clin Electrophysiol. 2024;10(4):654–666. 38385912 10.1016/j.jacep.2023.12.002

[102] Arkles J , Markman T , Trevillian R , et al. One‐year outcomes after stereotactic body radiotherapy for refractory ventricular tachycardia. Heart Rhythm. 2024;21(1):18–24. 37827346 10.1016/j.hrthm.2023.10.005

[103] Borzov E , Efraim R , Suleiman M , et al. Implementing stereotactic arrhythmia radioablation with STOPSTORM.eu consortium support: intermediate results of a prospective Israeli single‐institutional trial. Strahlenther Onkol 2025;201:126–134. doi:10.1007/s00066-024-02300-z 39283343 PMC11754307

[104] Bianchi S , Marchesano D , Magnocavallo M , et al. Magnetic resonance‐guided stereotactic radioablation for septal ventricular tachycardias. JACC Clin Electrophysiol. 2024;10(12):2569–2580. 39387741 10.1016/j.jacep.2024.08.008

[105] Das SK , Ryan T , Panettieri V , et al. Stereotactic arrhythmia radioablation for refractory ventricular tachycardia—The initial Australian experience. Heart Rhythm. 2025;22(8):e364‐e373. 39922405 10.1016/j.hrthm.2025.02.005

[106] Jiang SJ , Samson P , Cuculich P , et al. Stereotactic arrhythmia radiation therapy versus repeat catheter ablation for refractory ventricular tachycardia: 3‐year safety and efficacy outcomes. Int J Radiat Oncol Biol Phys 2025;22(8):e364‐e373. doi:10.1016/j.ijrobp.2025.09.006 41033611

[107] Klein EE , Hanley J , Bayouth J , et al. Task Group 142 report: quality assurance of medical acceleratorsa. Med Phys. 2009;36(9Part1):4197–4212. 19810494 10.1118/1.3190392

[108] Miften M , Mihailidis D , Kry SF , et al. Management of radiotherapy patients with implanted cardiac pacemakers and defibrillators: A Report of the AAPM TG‐203†. Med Phys. 2019;46(12):e757‐e788. 31571229 10.1002/mp.13838

[109] Miften M , Olch A , Mihailidis D , et al. Tolerance limits and methodologies for IMRT measurement‐based verification QA : Recommendations of AAPM Task Group No. 218 . Med Phys. 2018;45(4):e53‐e83. 29443390 10.1002/mp.12810

[110] Shah KD , Chang C‐W , Patel P , et al. A comparative dosimetric study of proton and photon therapy in stereotactic arrhythmia radioablation for ventricular tachycardia. ArXiv 2025. 10.1016/j.phro.2025.100807 PMC12274703 40687305

[111] van der Ree MH , Visser J , Planken RN , et al. Standardizing the cardiac radioablation targeting workflow: enabling semi‐automated angulation and segmentation of the ssHeart according to the American heart association segmented model. Adv Radiat Oncol. 2022;7(4):100928. 35387177 10.1016/j.adro.2022.100928 PMC8978276

[112] Morris E , Chin R , Wu T , Smith C , Nejad‐Davarani S , Cao M . ASSET: Auto‐Segmentation of the Seventeen SEgments for Ventricular Tachycardia Ablation in radiation therapy. Cancers (Basel). 2023;15(16). doi:10.1016/j.radonc.2024.110499 PMC10452457 37627090

[113] Hohmann S , Xie J , Eckl M , et al. Semi‐automated reproducible target transfer for cardiac radioablation ‐ A multi‐center cross‐validation study within the RAVENTA trial. Radiother Oncol. 2024;200:110499. 39242029 10.1016/j.radonc.2024.110499

[114] van der Pol LHG , Blanck O , Grehn M , et al. Auto‐contouring of cardiac substructures for Stereotactic arrhythmia radioablation (STAR): A STOPSTORM.eu consortium study. Radiother Oncol. 2024;202:110610. 39489426 10.1016/j.radonc.2024.110610

