# Supplementary material for: Stereotactic arrhythmia radioablation for refractory ventricular tachycardia: A narrative review and pooled analysis of clinical outcomes and treatment delivery approaches
Source: J Appl Clin Med Phys. 2026 May 12;27(5):e70622. doi: 10.1002/acm2.70622 (PMC13167255; doi:10.1002/acm2.70622)
Supplement: Supplementary file 1 — Supporting Information [file ACM2-27-e70622-s001.docx]

**Stereotactic Arrhythmia Radioablation for Refractory Ventricular Tachycardia: A Narrative Review and Pooled Analysis of Clinical Outcomes and Treatment Delivery Approaches**

Keyur D. Shah, PhD^1^, Chih-Wei Chang, PhD^1^, Sibo Tian, MD^1^, Pretesh Patel, MD^1^, Richard Qiu, PhD^1^, Shadab Momin, PhD^1^, Justin Roper, PhD^1^, Jun Zhou, PhD^1^,

Zhen Tian, PhD^2^ and Xiaofeng Yang, PhD^1*^

^1^Department of Radiation Oncology and Winship Cancer Institute, Emory University, Atlanta, GA

^2^Department of Radiation & Cellular Oncology, University of Chicago, Chicago, IL

*Corresponding to: xiaofeng.yang@emory.edu

**Running title:** STAR for Ventricular Tachycardia: Narrative Review & Pooled Analysis

**Manuscript Type:** Review Article

**Keywords:** Stereotactic Arrhythmia Radioablation (STAR), Stereotactic Body Radiation Therapy (SBRT), Ventricular Tachycardia (VT), Cardiac Radiosurgery, Noninvasive VT Ablation

**Funding:** This research is supported in part by the National Institutes of Health under Award Number R01CA272991, R01EB032680, R37CA272755 and U54CA274513.

**COI Statement:** All authors declare that they have no known conflicts of interest in terms of competing financial interests or personal relationships that could have an influence or are relevant to the work reported on this page.

**Author Contributions:** Keyur D. Shah: Conceptualization; Methodology; Data curation; Software; Formal analysis; Investigation; Visualization; Writing—original draft. Chih-Wei Chang: Writing—review & editing. Sibo Tian: Writing—review & editing. Pretesh Patel: Writing—review & editing. Richard Qiu: Writing—review & editing. Shadab Momin: Writing—review & editing. Justin Roper: Writing—review & editing. Jun Zhou: Writing—review & editing. Zhen Tian: Writing—review & editing. Xiaofeng Yang: Conceptualization; Supervision; Funding acquisition; Project administration; Writing—review & editing.
