# Supplementary material for: Stereotactic arrhythmia radioablation for refractory ventricular tachycardia: A narrative review and pooled analysis of clinical outcomes and treatment delivery approaches
Source: J Appl Clin Med Phys. 2026 May 12;27(5):e70622. doi: 10.1002/acm2.70622 (PMC13167255; doi:10.1002/acm2.70622)
Supplement: Supplementary file 2 — Supporting Information [file ACM2-27-e70622-s003.docx]

**Supplementary Materials**

**
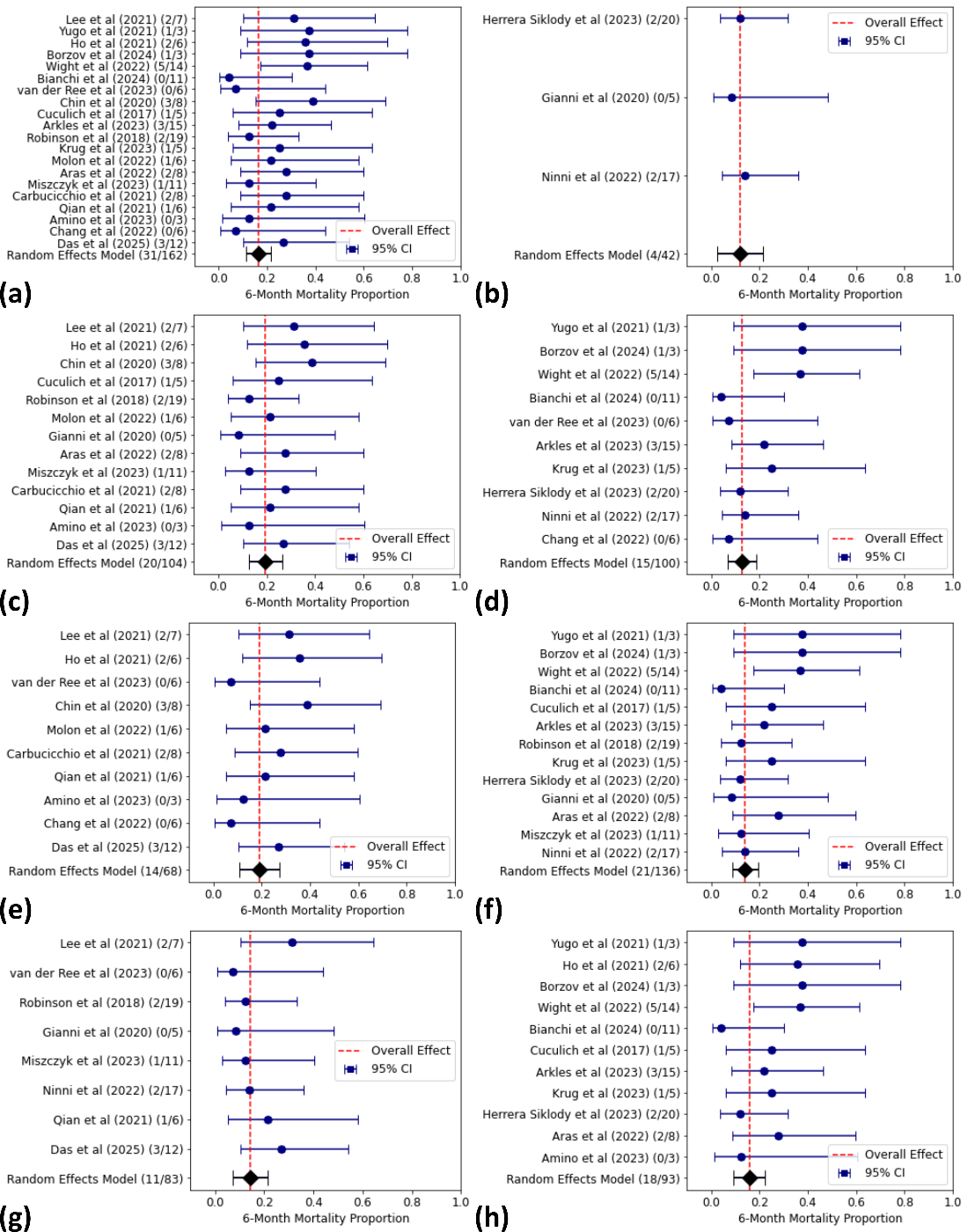
**

**Figure S1.** Forest plots depicting pooled mortality proportion 6 months following stereotactic arrhythmia radioablation (STAR), stratified by key subgroups. Each plot shows individual study estimates with 95% confidence intervals and an overall pooled estimate using a random-effects model. Studies are labeled with their reported VT reduction (%) and ordered chronologically. Subgroup comparisons include: **(A–B)** treatment modality — LINAC-based STAR **(A)** vs. CyberKnife **(B)**, **(C–D)** baseline left ventricular ejection fraction (LVEF) ≤ median **(C)** vs. > median **(D)**, **(E–F)** patient age ≤ median **(E)** vs. > median **(F)**, and **(G–H)** underlying cardiomyopathy — ischemic (ICM) **(G)** vs. non-ischemic (NICM) **(H)**.


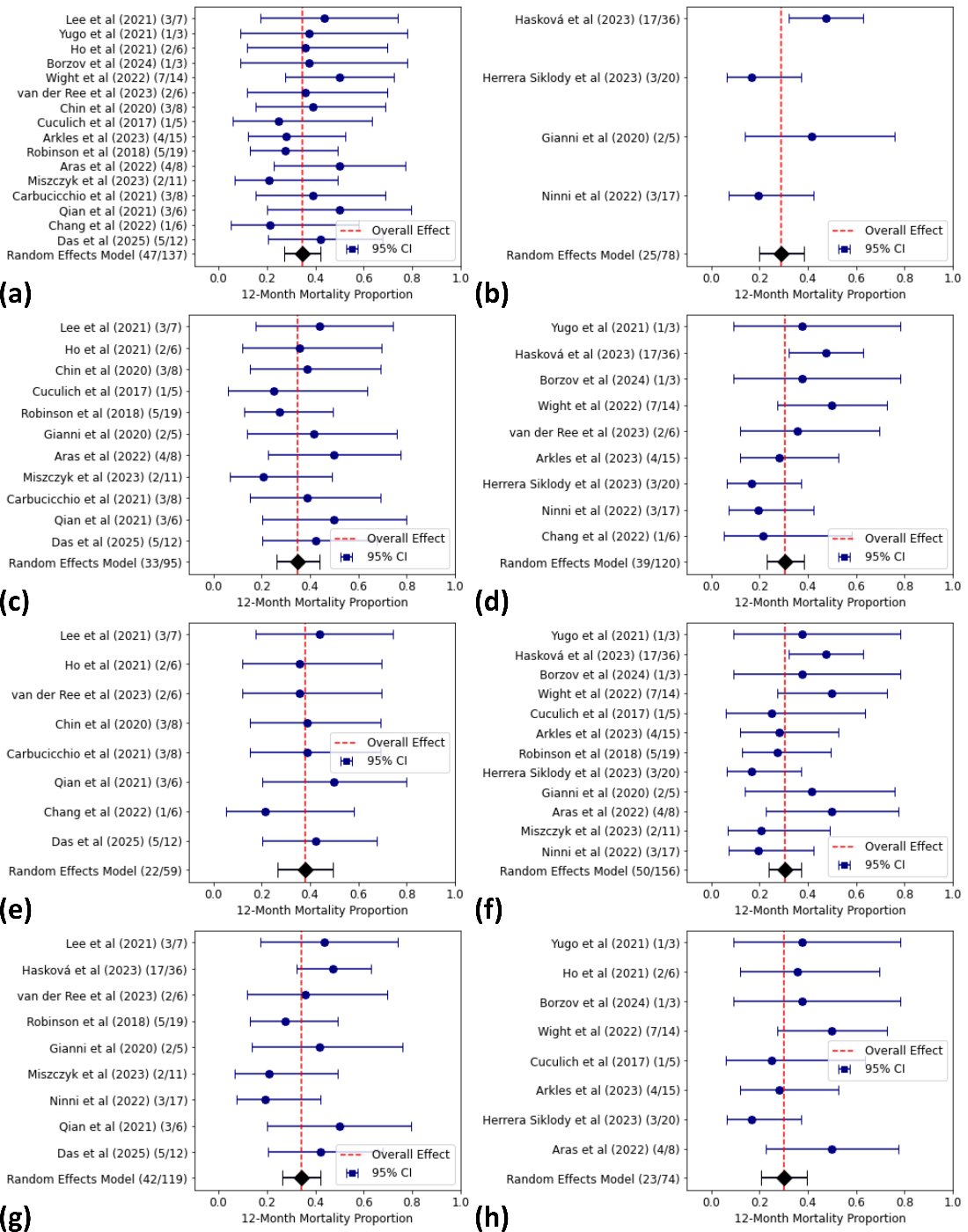


**Figure S2.** Forest plots depicting pooled mortality proportion 12 months following stereotactic arrhythmia radioablation (STAR), stratified by key subgroups. Each plot shows individual study estimates with 95% confidence intervals and an overall pooled estimate using a random-effects model. Studies are labeled with their reported VT reduction (%) and ordered chronologically. Subgroup comparisons include: **(A–B)** treatment modality — LINAC-based STAR **(A)** vs. CyberKnife **(B)**, **(C–D)** baseline left ventricular ejection fraction (LVEF) ≤ median **(C)** vs. > median **(D)**, **(E–F)** patient age ≤ median **(E)** vs. > median **(F)**, and **(G–H)** underlying cardiomyopathy — ischemic (ICM) **(G)** vs. non-ischemic (NICM) **(H)**.


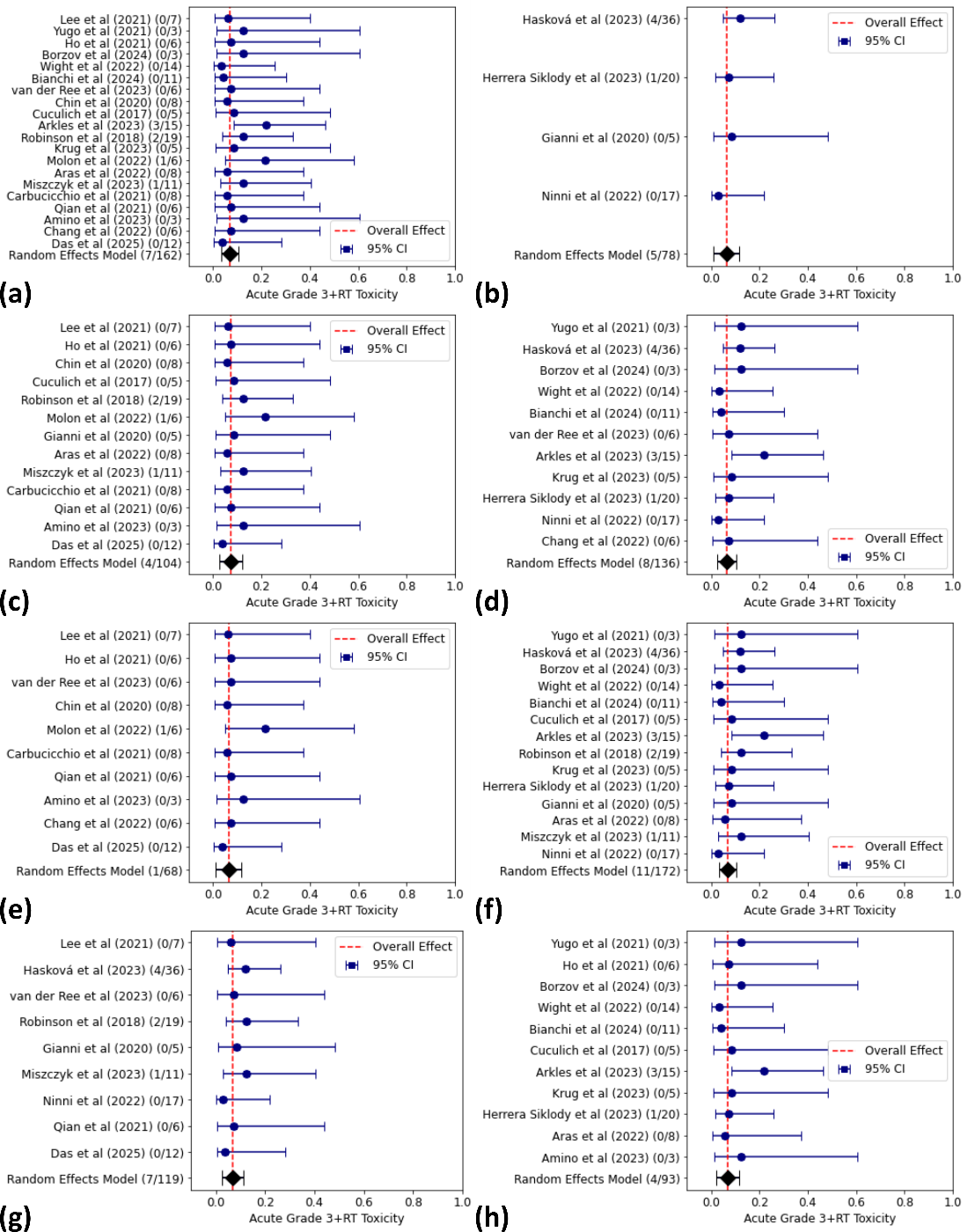


**Figure S3.** Forest plots depicting pooled acute adverse events following stereotactic arrhythmia radioablation (STAR), stratified by key subgroups. Each plot shows individual study estimates with 95% confidence intervals and an overall pooled estimate using a random-effects model. Studies are labeled with their reported VT reduction (%) and ordered chronologically. Subgroup comparisons include: **(A–B)** treatment modality — LINAC-based STAR **(A)** vs. CyberKnife **(B)**, **(C–D)** baseline left ventricular ejection fraction (LVEF) ≤ median **(C)** vs. > median **(D)**, **(E–F)** patient age ≤ median **(E)** vs. > median **(F)**, and **(G–H)** underlying cardiomyopathy — ischemic (ICM) **(G)** vs. non-ischemic (NICM) **(H)**.
