# Supplementary material for: Stereotactic arrhythmia radioablation for refractory ventricular tachycardia: A narrative review and pooled analysis of clinical outcomes and treatment delivery approaches
Source: J Appl Clin Med Phys. 2026 May 12;27(5):e70622. doi: 10.1002/acm2.70622 (PMC13167255; doi:10.1002/acm2.70622)
Supplement: Supplementary file 3 — Supporting Information [file ACM2-27-e70622-s002.docx]

**Figure Captions**

**Figure 1.** **PRISMA Flow Diagram of Study Selection Process.** Illustrates the selection process for included studies, detailing the number of records identified, screened, excluded, and ultimately included in this review. Reasons for exclusion are categorized, including unrelated topics, insufficient data, duplicates, and non-English publications. (*4 studies were added after independent citation analysis). Modified from Page et al ^25^

**Figure 2. Forest Plots Summarizing Pooled-Analysis Results for Mortality, VT Burden Reduction, and Acute Toxicity.** Forest plots displaying the pooled effect estimates and 95% confidence intervals (CI) for **(a)** 6-month mortality, **(b)** 12-month mortality, **(c)** acute grade 3+ toxicity rates within 90 days, and **(d)** VT reduction at 6 months. The red dashed line represents the overall effect, while individual blue points and bars represent study-specific estimates and their CIs. A random-effects model was used for pooled-analysis.

**Figure 3.** Forest plots depicting pooled VT burden reduction at 6 months following stereotactic arrhythmia radioablation (STAR), stratified by key subgroups. Each plot shows individual study estimates with 95% confidence intervals and an overall pooled estimate using a random-effects model. Studies are labeled with their reported VT reduction (%) and ordered chronologically. Subgroup comparisons include: **(A–B)** treatment modality — LINAC-based STAR **(A)** vs. CyberKnife **(B)**, **(C–D)** baseline left ventricular ejection fraction (LVEF) ≤ median **(C)** vs. > median **(D)**, **(E–F)** patient age ≤ median **(E)** vs. > median **(F)**, and **(G–H)** underlying cardiomyopathy — ischemic (ICM) **(G)** vs. non-ischemic (NICM) **(H)**.

**Table Captions**

**Table 1.** Summary of Preclinical Studies Investigating Stereotactic Arrhythmia Radioablation (STAR) and Particle Therapy for Cardiac Applications.

**Table 2.** Summary of Case Reports Investigating Stereotactic Arrhythmia Radioablation (STAR) for Ventricular Tachycardia (VT).

**Table 3.** Summary of Clinical Trials and Case Series Investigating Stereotactic Arrhythmia Radioablation (STAR) for Ventricular Tachycardia (VT). If the entry is a clinical trial, its trial name is reported in the Author column.

**Table 4**. Summary of Pooled-Analysis Results for Stereotactic Arrhythmia Radioablation (STAR) in Ventricular Tachycardia (VT): Outcomes include pooled estimates for mortality at 6 and 12 months, reduction in VT burden at 6 months, and grade 3+ adverse events within 90 days. Subgroup analyses evaluate variations by treatment modality (LINAC vs. CyberKnife), LVEF (≤ median vs. > median), patient age (≤ median vs. > median), and cardiomyopathy type (ICM vs. NICM). Results are presented as pooled effect estimates with 95% confidence intervals (CI), Cochran’s Q statistics, and heterogeneity (I²).
